# Incompatibility of Frequency Splitting and Spatial Localization: A Quantitative Analysis of Hegerfeldt’s Theorem

**DOI:** 10.1007/s00023-022-01215-8

**Published:** 2022-09-16

**Authors:** Felix Finster, Claudio F. Paganini

**Affiliations:** 1 Fakultät für Mathematik, Universität Regensburg, 93040 Regensburg, Germany; 2 Max Planck Institute for Gravitational Physics (Albert Einstein Institute), Am Mühlenberg 1, 14476 Potsdam, Germany

## Abstract

We prove quantitative versions of the following statement: If a solution of the $1+1$-dimensional wave equation has spatially compact support and consists mainly of positive frequencies, then it must have a significant high-frequency component. Similar results are proven for the $3+1$-dimensional wave equation.

## Introduction

The present paper provides a quantitative analysis of a problem that has been studied by different communities in different contexts. On the one hand, in *quantum theory* it is well known that spatial localization is incompatible with the Hamiltonian (i.e., the generator of time translations) to be bounded from below. This result, often referred to as *Hegerfeldt’s theorem*, means physically that a quantum system either propagates with infinite speed (thus violating causality), or else it must involve pair creation or annihilation processes as described by wave functions involving arbitrarily large negative frequencies.^1^ Hegerfeldt’s theorem has far-reaching consequences for our understanding of the interplay between locality and the distribution of energy in spacetime. To give a simple example, it explains why the Feynman propagator $G_\text {F}(x,y)$ (defined by the condition that “positive frequencies travel to the future” and “negative frequencies travel to the past”) cannot be causal but instead must have non-vanishing contributions for a large spacelike separation of *x* and *y*.

From the point of view of *harmonic analysis*, on the other hand, Hegerfeldt’s theorem can be regarded as an application of a classic theorem by F. and M. Riesz, a discussion of which can be found for example in [9,  Sect. I.1]. It constitutes a special case of an *annihilating pair of sets for the Fourier transform* as discussed in [10,  Sect. 1.2.1]. For related problems in harmonic analysis, see, for example, [21] but also [28], which contains a power-series argument similar to the one we develop in the course of our work in Sect. 4.4.

The proof of Hegerfeldt’s theorem (see [11] or the concise review in [5,  Theorem 3 in Sect. 4]) uses complex continuation and the Schwarz reflection principle. This method is general and elegant, but unfortunately it does not give quantitative information on the frequency splitting. The goal of the present paper is to prove quantitative versions of Hegerfeldt’s theorem. In order to make the paper accessible to a broader readership, we formulate the problem and our results purely in the language of hyperbolic partial differential equations (PDEs). From this perspective, Hegerfeldt’s theorem states that solutions of hyperbolic PDEs which have spatially compact support cannot be composed purely of positive (or similarly negative) frequencies. (A clear and detailed proof in the PDE language is given in [30,  Sect. 1.8] or [4,  Corollary 3.6].) The quantification we have in mind is the following: Suppose that at an initial time, a solution has compact support in a ball of radius *r*. What can one infer on the possible frequency distributions of the solution? In particular, how small can the component of negative (or similarly positive) frequency be?

Before making this question mathematically precise and stating our results, we give an overview of the literature on localization in quantum theory. The problem of localization in quantum theory has a long history (see, e.g., [31] for an overview of the early literature). It was on that backdrop that Hegerfeldt [11] proved in 1974 that a quantum mechanical system cannot be localized, or, if initially localized, will spread instantly and thus violate strong Einstein causality. Skagerstam [27] proved the same result with a different method. In particular, he provides an independent proof in the Heisenberg picture. A different attempt at localization using current density four vectors was pursued in [7, 8]. Hegerfeldt’s results were generalized by several authors [12, 16, 23]. In a series of later articles [13–15], Hegerfeldt discussed these results and their observational consequences in greater detail. Hegerfeldt’s theorem has applications to quantum theory in the context of causal localizations (see, e.g., [5, 6] and the references therein for more recent developments). In [15], Hegerfeldt addresses the question why the Dirac equation is not a counter example: The original result is based on the assumption that the Hamiltonian of the system is positive definite, which obviously is not the case for the Dirac Hamiltonian. The fact that localized solutions to the Dirac equation always contain contributions of positive and negative energy has been linked [14] to the insight from the field-theoretic perspective that an effective particle corresponds to a “dressed” state, i.e., that it is surrounded by a cloud of “virtual” particle-antiparticle pairs. The appearance of contributions of both positive and negative frequencies in a localized solution to the Dirac equation can be thought of as the PDE counterpart to this phenomenon.

In the PDE literature, questions similar to those considered in the context of localization in quantum theory were addressed in [19, 20, 25] in terms of *unique continuation theorems*, i.e., statements of the type that if a solution to a PDE of interest (namely the Schrödinger equation in [19] or the scalar wave equation in [20]) vanishes in an open region, then it vanishes everywhere, provided that one requires the solution to be in a suitable regularity class. Furthermore, see [2, 3] for related results on a Riemannian manifold and [25,  Sect. 13] for a discussion of similar results for the Schrödinger equation with a potential. It should be noted that, although these results are clearly related, the formulation of the PDE problem does not immediately translate to the formulation of the problem of localization in quantum mechanics. The PDE problem assumes the vanishing of a function in a certain domain, while the problem of localization in quantum mechanics assumes that the expectation value of a self-adjoint operator, which is associated with a certain spatial region, vanishes.

We now specify the mathematical problem and state our main results. For simplicity, we restrict attention throughout to the cases of the scalar wave equation in one and three spatial dimensions. But, as will become clear from our analysis, our methods also apply to other dimensions as well as to the Klein–Gordon equation. Moreover, our results immediately apply to the equations of higher spin (Maxwell, Dirac, Rarita–Schwinger, linearized gravity), simply because in Minkowski space, each component of a solution to these equations satisfies the scalar wave equation or Klein–Gordon equation.

In preparation, let us consider the following question:

(A) Assume that at some time $t_0$, a wave $\phi (t,x)$ is spatially supported inside a ball of radius *r*. Does this imply an a priori bound for the ratio
  1.1 $$\begin{aligned} \frac{E(\phi _+)}{E(\phi _-)} \end{aligned}$$
  of the energies of the components of positive and negative frequency? (For notational details, see Sect. 2.)

The answer to this question is *no*. Indeed, by making the absolute value of the frequencies of $\phi$ sufficiently large, one can make quotient (1.1) arbitrarily large or small (for more details see Sect. 3). But, turning this argument around, one concludes that if quotient (1.1) is small, then the wave should have significant high-frequency contributions. The goal of this paper is to quantify this statement by results of the following form:

### Theorem 1.1

Let $\phi (t,x)$ be a solution of the scalar wave equation which at some time $t_0$ is supported inside a ball of radius $r>0$,

$$\begin{aligned} {{\,\mathrm{supp}\,}}\phi (t_0, .) \subset B_r(0) {.} \end{aligned}$$

Assume that the inequality

$$\begin{aligned} E(\phi _-) \le \varepsilon ^2\, E(\phi ) \end{aligned}$$

holds for some $\varepsilon \in (0,1]$. Then, there is an a priori estimate for the momentum distribution of $\phi$ of the form

1.2 $$\begin{aligned} \big |k\,\hat{\phi }(k) \big | + \big | \partial _t \hat{\phi }(k) \big | \le R\big (\varepsilon , r \,|k| \big ) \,\sqrt{r\,E(\phi )}{.} \end{aligned}$$

Here, $\hat{\phi }$ denotes the spatial Fourier transform (for details see again Sect. 2).

The dispersion relation for the wave equation yields that frequency and momentum coincide up to a sign. Therefore, inequality (1.2) also tells us about the frequency distribution. By direct computation or using a dimensional argument, one readily verifies that inequality (1.2) is scaling invariant. With this in mind, we can always restrict attention to the case $r=1$ of a unit ball. We shall derive several closed expressions for the function *R* (see Theorems 4.10 and 4.13 and Corollary 4.25, where we always set $\omega =|k|$). All these expressions vanish in the limit $\varepsilon \searrow 0$,

$$\begin{aligned} \lim _{\varepsilon \searrow 0} R\big (\varepsilon , |k| \big ) = 0 \qquad \text {for all } k{,} \end{aligned}$$

as needed for the correspondence to Hegerfeldt’s theorem. If $\varepsilon$ is positive and small, inequality (1.2) implies that $\hat{\phi }(k)$ is small unless |*k*| is large. This can be understood as a form of unique continuation, in the sense that, assuming the Fourier transform to have relatively small $L^2$ mass for negative frequencies, we show that the absolute value of the Fourier transform has to be small for small positive frequencies. For partial differential equations, unique continuation theorems of a similar spirit can be found in [17, 29]. There are also related unique continuation results for the Hilbert transform as given for example in [1, 26]. However, in contrast to these results, it is a specific feature of our method that we aim at getting uniform estimates for all values of the two parameters $\varepsilon$ and *k*. It is one of our main goals to unravel the functional dependence on these two parameters.

We begin with simple but rough bounds that give a good first understanding of the underlying mechanism and might be sufficient for some applications. In the subsequent, more technical parts of the paper we show that our estimate of the series expansion of the Fourier transform is a solution of a Goursat problem, and employing stationary phase techniques will give rise to significantly improved upper bounds.

In contrast to Hegerfeldt’s approach, our methods do not rely on complex analysis. Instead, working with Legendre polynomials, we derive estimates for each Taylor coefficient of the Fourier transform. From that, we infer explicit upper bounds for the Fourier transform at low frequencies. Hegerfeldt’s result is obtained in the present considerations by the fact that if we take the limiting case when the compactly supported solution is supported only in the positive frequencies, then the Fourier transform vanishes everywhere, and thus the function itself is trivial.

We finally note that we expect that our methods and results apply in a much more general setting. One possible extension is to higher dimensions, as we here illustrate by deriving estimates for every angular momentum mode of the wave equation in three spatial dimensions. Moreover, the assumption of compact support could probably be replaced by suitable decay assumptions of the initial data. Finally, our results should apply to massive equations, to situations in the presence of external potentials and to equations in curved spacetimes. Another possible extension would be to consider different decompositions of momentum space into two subsets which generalize the notions of positive and negative frequencies. However, these extensions and generalizations go beyond the scope of the present paper.

The paper is structured as follows. In Sect. 2, we introduce the mathematical setup and fix our notation. In Sect. 3, we discuss a simple example. The main part of the paper is concerned with the one-dimensional wave equation (Sect. 4). After recalling a simple pointwise estimate of the Fourier transform (Sect. 4.1), we expand the Fourier transform in a power series (Sect. 4.2) and derive simple estimates of the Taylor coefficients in terms of the energy (Sect. 4.3). In order to derive refined estimates, we decompose the Fourier series into a polynomial and the remainder. The coefficients of the polynomial are bounded using $L^2$-estimates together with properties of Legendre polynomials (Sect. 4.4), whereas the remainder can be treated with the simple estimates (Sect. 4.5). This gives improved estimates of all Taylor coefficients (see Proposition 4.7) which give rise to estimate the energy distribution of the initial data in terms of a series $g(\varepsilon , \omega )$ (see Proposition 4.8 in Sect. 4.6). We proceed with a few simple estimates of this series (Sects. 4.7 and 4.8), which might be sufficient for future applications and are addressed more toward the theoretical physics community.

The key for getting better estimates of this series is the observation that, as a function of $\varepsilon$ and $\omega$, the series can be transformed to a solution of a characteristic initial value problem (Goursat problem) for the $1+1$-dimensional Klein–Gordon equation (Sect. 4.9). After bringing the initial data into a more explicit form (Sect. 4.10), we can solve the Goursat problem with the help of the Klein–Gordon Green’s operator and its representation in momentum space to obtain a contour integral (Sect. 4.11). This contour integral can be estimated with a saddle-point approximation and rigorous error bounds (Sect. 4.12). It remains to integrate over two parameters which came up in our constructions: the spatial momentum *k* (Sect. 4.13) and the parameter *s* used for the construction of the initial data (Sect. 4.14). We thus obtain the improved estimate for $g(\omega )$ in Theorem 4.24. This section contains a number of interesting technical results and is addressed more at the mathematical community. Finally, in Sect. 5 we extend the results to each angular mode of the $(3+1)$-dimensional wave equation (see Theorem 5.8). Appendix provides an alternative derivation of an integral representation of the solutions of the Goursat problem given in Sect. 4.9.

## Preliminaries

### Fourier Transform

We recall a well-known result, which is an immediate consequence of the Paley–Wiener theorem (see [32,  Sect. VI.4] or [24,  Theorem IX.11]).

#### Lemma 2.1

Let $\phi \in C^\infty _0(B_1(0))$ be a smooth real- or complex-valued function with compact support in the interval $(-1,1) \subset \mathbb {R}$. Then, its Fourier transform^2^

2.1 $$\begin{aligned} \hat{\phi }(k) = \int _{B_1} \phi (x) \, e^{-i kx}\, \mathrm{d}x \end{aligned}$$

can be represented as a power series

2.2 $$\begin{aligned} \hat{\phi }(k) = \sum _{n=0}^\infty c_n\, k^n {,} \end{aligned}$$

with coefficients $(c_n)_{n\in \mathbb {N}_0}$ bounded by

2.3 $$\begin{aligned} |c_n|&\le \frac{\sqrt{2}}{n!}\, \Vert \phi \Vert _{L^2(B_1)} \end{aligned}$$

2.4 $$\begin{aligned} |c_n|&\le \frac{\sqrt{2}}{(n+1)!}\, \Vert \partial _x \phi \Vert _{L^2(B_1)} {.} \end{aligned}$$

#### Proof

Differentiating (2.1), we obtain

$$\begin{aligned} \big | \hat{\phi }^{(n)}(k) \big | \le \bigg | \int _{B_1} (-ix)^n \,\phi (x) \, e^{-i kx}\, \mathrm{d}x \bigg | \le \int _{B_1} \big |\phi (x)\big | \, \mathrm{d}x \le \sqrt{2}\, \Vert \phi \Vert _{L^2(B_1)} {.} \end{aligned}$$

In particular, setting $k=0$ we obtain

$$\begin{aligned} \big |c_n\big |\, n! = \big | \hat{\phi }^{(n)}(0) \big | \le \sqrt{2}\, \Vert \phi \Vert _{L^2(B_1)} {,} \end{aligned}$$

giving the desired bound (2.3). Moreover, we conclude that the Taylor series converges absolutely.

In order to derive (2.4), we consider similarly the Fourier transform of the derivative of $\phi (x)$ to obtain

$$\begin{aligned} i k\, \hat{\phi }(k) = \sum _{n=1}^\infty d_n\, k^n \qquad \text {with} \qquad |d_n| \le \frac{\sqrt{2}}{n!}\, \Vert \partial _x\phi \Vert _{L^2(B_1)} {.} \end{aligned}$$

Comparing the last equation with (2.2), one sees that $c_n=-i d_{n+1}$, giving (2.4). $\square$

This estimate shows in particular that $\hat{\phi }(k)$ is real analytic.

### Green’s Operators and the Causal Fundamental Solution

The proof of our main theorem is based on estimates of a solution of the Klein–Gordon equation in $1+1$ dimensions (for details see Sect. 4.9). We now recall the basics on Green’s operators needed for this analysis. The Klein–Gordon equation for a wave $\phi$ of mass $m \ge 0$ reads

$$\begin{aligned} \big ( \partial _t^2 - \partial _x^2 + m^2 \big ) \, \phi (t,x) = 0 {.} \end{aligned}$$

Green’s kernels are distributional solutions of this equation with a $\delta$-distribution as inhomogeneity. More precisely, they are defined by the equation

2.5 $$\begin{aligned} \big ( \partial _t^2 - \partial _x^2 + m^2 \big ) \, S_{m^2}(t,x) = -\delta (t)\, \delta (x){.} \end{aligned}$$

The Green’s operator $S_{m^2}$ is the corresponding integral operator defined by

2.6 $$\begin{aligned} (S \phi )(t,x) := \int _{\mathbb {R}^2} S_{m^2}(t-t', x-x')\, \phi (t',x')\, \mathrm{d}t'\, \mathrm{d}x' {.} \end{aligned}$$

We now compute the Green’s kernel with Fourier methods. Taking the Fourier transform of the Green’s kernel,

$$\begin{aligned} S_{m^2}(t,x) = \int _{\mathbb {R}^2} \frac{\mathrm{d}\omega \, \mathrm{d}k}{(2 \pi )^2}\, \hat{S}_{m^2}(\omega , k)\, e^{-i \omega t+ i k x} {,} \end{aligned}$$

the differential equation (2.5) reduces to the algebraic equation

$$\begin{aligned} (\omega ^2 - k^2-m^2)\, \hat{S}(\omega ,k) = 1{.} \end{aligned}$$

When solving this equation, one must treat the zeros of the function $\omega ^2 - k^2-m^2$ with a suitable deformation in the complex plane. For our purposes, it is useful to choose

2.7 $$\begin{aligned} \begin{aligned} \hat{S}_{m^2}^\vee (\omega ,k)&= \lim _{\varepsilon \searrow 0} \frac{1}{\omega ^2-k^2-m^2 - i \varepsilon \omega } \\ \hat{S}_{m^2}^\wedge (\omega ,k)&= \lim _{\varepsilon \searrow 0} \frac{1}{\omega ^2-k^2-m^2 + i \varepsilon \omega } \end{aligned} \end{aligned}$$

where the limit $\varepsilon \searrow 0$ is taken in the distributional sense. The resulting Fourier transform can be computed explicitly with residues. Indeed, carrying out the $\omega$-integral by closing the contour in the upper (lower) half plane if $t<0$ (respectively, $t>0$), we get

$$\begin{aligned}&S^\wedge _{m^2}(t,x) = \lim _{\varepsilon \searrow 0} \int _{\mathbb {R}^2} \frac{\mathrm{d}\omega \, \mathrm{d}k}{(2 \pi )^2}\, \frac{1}{\omega ^2-k^2-m^2 + i \varepsilon \omega }\, e^{-i \omega t+ i k x} \\&= \lim _{\varepsilon \searrow 0} \int _{\mathbb {R}^2} \frac{\mathrm{d}\omega \, \mathrm{d}k}{(2 \pi )^2}\, \bigg ( \frac{1}{\omega - \sqrt{k^2+m^2} + i \varepsilon } - \frac{1}{\omega +\sqrt{k^2+m^2} + i \varepsilon } \bigg ) \, \frac{e^{-i \omega t+ i k x}}{2\,\sqrt{k^2+m^2}} \\&= \Theta (t)\, \frac{(-2 \pi i)}{(2 \pi )^2} \int _{-\infty }^\infty \frac{\mathrm{d}k}{2\,\sqrt{k^2+m^2}} \,\Big ( e^{-i \sqrt{k^2+m^2}\, t} - e^{i \sqrt{k^2+m^2}\, t} \Big ) \, e^{i k x} \\&= -\Theta (t)\, \frac{1}{\pi } \int _{0}^\infty \frac{\mathrm{d}k}{\sqrt{k^2+m^2}} \, \sin \Big (\sqrt{k^2+m^2}\, t \Big )\,\cos (kx) \\&= \left\{ \begin{array}{c} \omega ^2 = k^2+m^2 \\ \displaystyle \frac{\mathrm{d}k}{\omega } = \frac{\mathrm{d}\omega }{k} \end{array} \right\} = -\Theta (t)\, \frac{1}{\pi } \int _{m}^\infty \frac{\mathrm{d}\omega }{\sqrt{\omega ^2-m^2}} \, \sin \big (\omega t \big )\,\cos \Big ( \sqrt{\omega ^2-m^2} \,x\Big ) \end{aligned}$$

where $\Theta$ is the Heaviside function. The obtained integral is well defined as an improper Riemann integral. In order to compute it, it is most convenient to make use of Lorentz invariance, making it possible to restrict attention to the case $x=0$. In this case, the Fourier integral can be carried out using Bessel functions (see [22,  Eq. 10.9.12])

$$\begin{aligned} \int _{m}^\infty \frac{\mathrm{d}\omega }{\sqrt{\omega ^2-m^2}} \, \sin \big (\omega t \big ) = \int _{1}^\infty \frac{\mathrm{d}\sigma }{\sqrt{\sigma ^2-1}} \, \sin \big (\sigma \,(m t) \big ) = \frac{\pi }{2}\, J_0(mt) {,} \end{aligned}$$

giving the explicit formula

2.8 $$\begin{aligned} S^\wedge _{m^2}(t,x) = -\frac{1}{2}\,\Theta (t)\, \Theta \big (t^2-x^2 \big )\, J_0 \Big ( m\, \sqrt{t^2-x^2} \Big ) {.} \end{aligned}$$

This Green’s kernel vanishes unless the point (*t*, *x*) lies in future light cone centered at the origin. As a consequence, in the Green’s operator (2.6) the function $\phi$ enters only inside the past light cone centered at (*t*, *x*). This is the reason why $S^\wedge _{m^2}$ is referred to as the *retarded Green’s operator*. Similarly, the Green’s kernel $S^\vee _{m^2}(t,x)$ is computed by

2.9 $$\begin{aligned} S^\vee _{m^2}(t,x) = -\frac{1}{2}\,\Theta (-t)\, \Theta \big (t^2-x^2 \big )\, J_0 \Big ( m\, \sqrt{t^2-x^2} \Big ) {,} \end{aligned}$$

giving rise to the *advanced Green’s operator* $S^\vee _{m^2}$.

We finally introduce the *fundamental solution* $K_{m^2}$ by

2.10 $$\begin{aligned} \begin{aligned} K_{m^2}(t,x) \;&:= \frac{1}{2 \pi i} \,\big ( S_{m^2}^\vee - S_{m^2}^\wedge \big )(t,x) \\&= -\frac{i}{4 \pi }\,\epsilon (t)\, \Theta \big (t^2-x^2 \big )\, J_0 \Big ( m\, \sqrt{t^2-x^2} \Big ) \end{aligned} \end{aligned}$$

where $\epsilon$ is the sign function. Being composed of the difference of the advanced and retarded Green’s kernels, the kernel of the fundamental solution satisfies the homogeneous Klein–Gordon equation,

2.11 $$\begin{aligned} \big ( \partial _t^2 - \partial _x^2 + m^2 \big ) \, K_{m^2}(t,x) = 0 {.} \end{aligned}$$

For this reason, the fundamental solution can be used to construct solutions of the Klein–Gordon and wave equations. The causal fundamental solution has the Fourier representation

2.12 $$\begin{aligned} K_{m^2}(t,x) = \int _{\mathbb {R}^2} \frac{\mathrm{d}\omega \, \mathrm{d}k}{(2 \pi )^2}\, \delta \big ( \omega ^2-k^2-m^2 \big )\, \epsilon (\omega ) \, e^{-i \omega t+ i k x} {.} \end{aligned}$$

Here, the fact that the integrand is supported on the mass shell $\omega ^2+k^2=m^2$ can be understood immediately from the fact that $K_{m^2}$ satisfies the Klein–Gordon Eq. (2.11). The detailed form of this integrand can be derived from (2.10) and (2.7) by using the distributional relation

$$\begin{aligned} \lim _{\varepsilon \searrow 0} \left( \frac{1}{x - i \varepsilon } - \frac{1}{x + i \varepsilon } \right) =\, 2 \pi i \, \delta (x) \end{aligned}$$

to obtain

$$\begin{aligned} S_{m^2}^\vee (\omega , k) - S_{m^2}^\wedge (\omega , k)&= \lim _{\varepsilon \searrow 0} \left[ \frac{1}{\omega ^2 - k^{2}-m^{2}-i \varepsilon \omega } - \frac{1}{\omega ^2 - k^{2}-m^{2}+i \varepsilon \omega } \right] \\&= \lim _{\varepsilon \searrow 0} \left[ \frac{1}{\omega ^2 - k^{2}-m^{2}-i \varepsilon } - \frac{1}{\omega ^2 - k^{2}-m^{2}+i \varepsilon } \right] \epsilon (\omega ) \\&= 2 \pi i\, \delta (\omega ^2 - k^2 -m^{2})\, \epsilon (q^{0}) {.} \end{aligned}$$

Alternatively, this relation can also be derived by direct computation of the Fourier integral in (2.12).

In the massless case $m=0$, we obtain the corresponding Green’s kernels and the fundamental solution of the wave equations. Using that $J_0(0)=1$, we get the simple formulas

2.13 $$\begin{aligned} S^\wedge _0(t,x)&= -\frac{1}{2}\,\Theta (t)\, \Theta \big (t^2-x^2 \big ) \end{aligned}$$

2.14 $$\begin{aligned} S^\vee _0(t,x)&= -\frac{1}{2}\,\Theta (-t)\, \Theta \big (t^2-x^2 \big ) \end{aligned}$$

2.15 $$\begin{aligned} K_0(t,x)&= -\frac{i}{4 \pi } \, \epsilon (t) \,\Theta \big (t^2-x^2 \big ) \end{aligned}$$

where $\epsilon$ is again the sign function.

## A Simple Example

The following example is intended to give the reader a first idea of the problem analyzed in this paper. In particular, the simple arguments presented in this section explain why the answer to the naive question (A) on page 3 is no.

Let  be a compactly supported test function in $1+1$-dimensional Minkowski spacetime . For notational clarity, we denote points of Minkowski space in boldface, i.e., ${\textbf{x}}=({\textbf{x}}^0, {\textbf{x}}^1)=(t,x)$ and ${\textbf{p}}=({\textbf{p}}^0, {\textbf{p}}^1=k)$. We again let $K_0$ be the causal fundamental solution (2.15). Then, the function3.1is a solution of the scalar wave equation which is smooth and has spatially compact support. Taking the Fourier transform in space and time, the convolution in (3.1) becomes a multiplication in momentum space, i.e.,

3.2 $$\begin{aligned} \phi ({\textbf{x}}) = \int _{\mathbb {R}^{2}} \frac{\mathrm{d}^{2}{\textbf{p}}}{(2 \pi )^{2}}\, \hat{K}_0({\textbf{p}})\, \hat{f}({\textbf{p}})\, e^{-i \,\langle {\textbf{p}}, {\textbf{x}} \rangle } \end{aligned}$$

where $\langle .,. \rangle$ is the Minkowski inner product. Using (2.12), the distribution $\hat{K}_0$ is given by

$$\begin{aligned} \hat{K}_0({\textbf{p}}) = \delta \big ( ({\textbf{p}}^0)^2 -({\textbf{p}}^1)^2 \big )\, \epsilon \big ( {\textbf{p}}^0 \big ) {.} \end{aligned}$$

We decompose the solution into the components of positive and negative frequencies by setting

3.3 $$\begin{aligned} \phi _\pm ({\textbf{x}}) = \int _{\mathbb {R}^{2}} \frac{\mathrm{d}^{2}p}{(2 \pi )^{2}}\, \Theta (\pm {\textbf{p}}_0)\, \hat{K}_0({\textbf{p}})\, \hat{f}({\textbf{p}})\, e^{-i \,\langle {\textbf{p}}, {\textbf{x}} \rangle } \end{aligned}$$

and denote their energies by

$$\begin{aligned} E\big (\phi _\pm \big ) := \frac{1}{2} \int _{-\infty }^\infty \Big ( \big |\partial _t \phi _\pm (t,x)\big |^2 + \big |\partial _x \phi _\pm (t,x)\big |^2 \Big )\, \mathrm{d}x {.} \end{aligned}$$

Clearly, these energies are time independent due to energy conservation.

We now answer question (A) on page 3:

### Proposition 3.1

For any $\varepsilon >0$, there is a smooth solution $\phi ({\textbf{x}})$ with spatially compact support of the wave equation in $(1+1)$-dimensional Minkowski space with the property that

$$\begin{aligned} \frac{E(\phi _-)}{E(\phi _+)} \le \varepsilon ^2 {.} \end{aligned}$$

### Proof

Given , in (3.1) we consider the family of test functions

$$\begin{aligned} f_\zeta ({\textbf{x}}) := f({\textbf{x}})\, \exp \big (-i \zeta \, ({\textbf{x}}^0+{\textbf{x}}^1) \big ) {,} \end{aligned}$$

where $\zeta$ is a positive parameter. For convenience, the test function *f* is chosen such that $\max _{\mathbb {R}^{2}}(\hat{f})=\hat{f}(0,0)$. Taking the Fourier transform, the multiplication by a plane wave translates into a shift of the argument, i.e.,

$$\begin{aligned} \hat{f}_\zeta ({\textbf{p}}) = \hat{f}\big ({\textbf{p}}^0 - \zeta , {\textbf{p}}^1 +\zeta \big ) {.} \end{aligned}$$

We now consider the corresponding family of solutions $\phi _\zeta$ in (3.2).

**Fig. 1:**
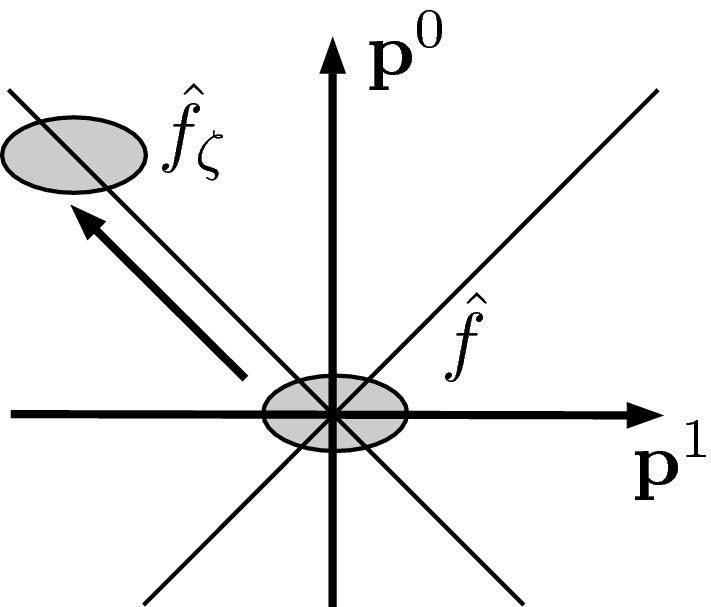
Shifting $\hat{f}_\zeta$ in momentum space. The shaded region indicates the neighborhood around the maximum of $\hat{f}_\zeta$, outside of which $\hat{f}_\zeta$ decays rapidly

By increasing $\zeta$, the function $\hat{f}_\zeta$ is shifted parallel to the light cone toward higher positive frequencies (Fig. 1) with $\max _{\mathbb {R}^{2}} \hat{f}_\zeta =\hat{f}(\zeta ,-\zeta )$. As a consequence, the energy $E(\phi _{\zeta , +})$ of the positive-frequency contribution is bounded from below. Furthermore, since $f({\textbf{x}})$ is smooth, its Fourier transform $\hat{f}$ decays rapidly. As a consequence, $\hat{\phi }_{\zeta , -}$ as well as its energy $E(\phi _{\zeta ,-})$ tend to zero rapidly in $\zeta$. Hence,

$$\begin{aligned} \lim _{\zeta \rightarrow \infty } \frac{E(\phi _{ \zeta ,-})}{E(\phi _{\zeta ,+})} = 0 {,} \end{aligned}$$

concluding the proof. $\square$

This example can be made more quantitative. In order to get a good example for testing our estimates, we want to choose a compactly supported function of one variable whose Fourier transform decays as fast as possible near infinity. As proven in [18,  Theorem in Sect. 1.5], there is a non-trivial, compactly supported function *g* whose Fourier transform is bounded by

3.4 $$\begin{aligned} |\hat{g}(k)| \le \exp \Big ( -\frac{|k|}{1 + \log ^2 |p|} \Big ) {.} \end{aligned}$$

This “almost exponential” decay near infinity is optimal in the sense that there is no compactly supported function *g* with (see [18,  Theorem in Sect. 1.1])

$$\begin{aligned} |\hat{g}(k)| \le \exp \Big ( -\frac{|k|}{1 + \log |p|} \Big ) {.} \end{aligned}$$

We choose

$$\begin{aligned} f({\textbf{x}}) = g \big ({\textbf{x}}^0 \big ) \, g\big ({\textbf{x}}^1 \big ) \end{aligned}$$

with *g* satisfying (3.4). For this choice of *g*, we can compute the energies of the corresponding solutions $\phi _\zeta$ in (3.2) and (3.3) as well as their spatial Fourier transforms (2.1) explicitly. A straightforward calculation yields

3.5 $$\begin{aligned} \big | k\, \hat{\phi }_{\zeta , +}(k) \big |&\le |k| \, \exp \bigg ( -\frac{\big | \zeta -|k| \big |}{1 + \log ^2 \big | \zeta - |k| \big | } \bigg ) \end{aligned}$$

3.6 $$\begin{aligned} \big | k\, \hat{\phi }_{\zeta , -}(k) \big |&\le |k| \, \exp \bigg ( -\frac{\zeta +|k|}{1 + \log ^2 \big | \zeta + |k| \big | } \bigg ) \end{aligned}$$

3.7 $$\begin{aligned} E(\phi _\zeta )&\sim \zeta ^2 \end{aligned}$$

3.8 $$\begin{aligned} E\big ( \phi _{\zeta ,-} \big )&\lesssim \int _0^\infty \omega ^2 \, \exp \bigg ( -\frac{2\,(\zeta + \omega )}{1 + \log ^2 (\omega +\zeta ) } \bigg ) \, \mathrm{d}\omega \nonumber \\&\lesssim \big ( 1 + \log ^2 \zeta )^3 \, \exp \bigg ( -\frac{2 \zeta }{1+\log ^2 \zeta } \bigg ) {.} \end{aligned}$$

Hence,

3.9 $$\begin{aligned} \varepsilon := \sqrt{\frac{E\big ( \phi _{\zeta ,-} \big )}{E(\phi _\zeta )}} \lesssim \frac{( 1 + \log ^2 \zeta )^{\frac{3}{2}}}{\zeta } \, \exp \bigg ( -\frac{\zeta }{1+\log ^2 \zeta } \bigg ) {.} \end{aligned}$$

Combining the above inequalities, one sees that for fixed *k* and small $\varepsilon$ (i.e., for large $\zeta$), in the above example the function *R* in (1.2) tends to zero in $\varepsilon$ slightly faster than linearly. Such a bound of $\hat{\phi }_\pm (k)$ in terms of $\varepsilon$ holds as long as the exponential in (3.5) is small, i.e., as long as $|k| \lesssim \zeta$. Inverting (3.9) asymptotically for large $\zeta$, one finds that $\zeta \sim -\log \varepsilon$. Therefore, the interval for |*k*| on which our improved estimate applies grows logarithmically in $\varepsilon$.

These qualitative findings will be reproduced by our estimates. Indeed, we shall see that for small *k* and $\varepsilon$, the function *R* in (1.2) scales like $R \sim \varepsilon ^\frac{2}{3}$ (see Proposition 4.8), which is consistent with the slightly faster than linear decay in $\varepsilon$ in the above example. Moreover, the logarithmic growth in $\varepsilon$ of the interval $|k| \in [0, \zeta ]$ also appears in our refined estimates (see, e.g., Proposition 4.21, where the region **(A)** is determined by inequality (4.68) with $k=\sqrt{2b}$ and $\lambda$, *a* and *b* as defined by (4.44) and (4.28) with $s=1$).

Although the methods used in this example give a good first understanding, it seems impossible to use them for proving Theorem 1.1. One reason is that the methods for analyzing the decay of Fourier transforms of compactly supported functions (see [18] for a good survey) do not give precise estimates. Another reason is that in (3.2) the function $\hat{f}_\zeta$ is multiplied by a distribution supported on the mass cone. As a consequence, results on the decay of two-dimensional Fourier transforms do not seem suitable for analyzing solutions of the wave equation.

## The $1+1$-Dimensional Case

In this section, we give a detailed analysis of the properties of solutions to the wave equation with spatially compact support in $1+1$-dimensional Minkowski space in the limiting case when the quotient $E(\phi _-)/E(\phi _+)$ is small. In particular, we shall derive an upper bound for the Fourier transform of such solutions for small frequencies.

We consider the Cauchy problem for the scalar wave equation with smooth initial data supported inside the unit ball $B_1=(-1,1)$,

4.1 $$\begin{aligned} \left\{ \begin{array}{l} (\partial _t^2 - \partial _x^2) \phi (t,\textbf {x}) = 0 \\ \phi |_{t=0} = \phi _0 \in C^\infty _0(B_1) {,} \qquad \partial _t \phi |_{t=0} = \phi _1 \in C^\infty _0(B_1) {.} \end{array} \right. \end{aligned}$$

We denote the energy of the solution by

4.2 $$\begin{aligned} E(\phi ) := \frac{1}{2} \int _{B_1} \Big ( \big |\partial _t \phi (0,x) \big |^2 + \big |\partial _x \phi (0,x) \big |^2 \Big )\, \mathrm{d}x {.} \end{aligned}$$

It is useful to take the Fourier transform of the spatial variable, again using the notation and conventions in (2.1). A direct computation yields

$$\begin{aligned} \hat{\phi }(t,k) = \hat{\phi }_+(t,k) + \hat{\phi }_-(t,k) \end{aligned}$$

with

4.3 $$\begin{aligned} \hat{\phi }_\pm (t,k) := \frac{1}{2} \, e^{\mp i \omega t}\Big ( \hat{\phi }_0(k) \pm \frac{i}{\omega }\, \hat{\phi }_1(k) \Big ) {,} \end{aligned}$$

where $\omega \ge 0$ denotes the absolute value of the frequency, i.e.,

4.4 $$\begin{aligned} \omega = \omega (k) := |k| {.} \end{aligned}$$

The solutions $\phi _\pm$ can be understood as the components of positive and negative frequency, respectively. This splitting is analogous to the splitting into plus- and minus-functions in [10,  p. 16]. Using Plancherel’s theorem, energy (4.2) can also be expressed as an integral in momentum space.

### Lemma 4.1

Energy (4.2) can be written as

4.5 $$\begin{aligned} E(\phi ) = E(\phi _+) + E(\phi _-) \qquad \text {with} \qquad E(\phi _\pm ) := \int _{-\infty }^\infty \frac{\mathrm{d}k}{2 \pi } \;\omega ^2\,\big |\hat{\phi }_\pm (k)\big |^2 {.}\nonumber \\ \end{aligned}$$

### Proof

A direct computation using Plancherel’s theorem yields

$$\begin{aligned} E(\phi )&= \frac{1}{2} \int _{-\infty }^\infty \frac{\mathrm{d}k}{2 \pi } \,\Big ( \omega ^2\, \big |\hat{\phi }_0(k)\big |^2 + \big |\hat{\phi }_1(k) \big |^2 \Big ) \\&= \int _{-\infty }^\infty \frac{\mathrm{d}k}{2 \pi } \;\omega ^2\,\Big ( \big |\hat{\phi }_+(t, k)\big |^2 + \big |\hat{\phi }_-(t, k)\big |^2 \Big ) {,} \end{aligned}$$

giving the result. $\square$

We now enter the proof of Theorem 1.1 in different versions (see Lemma 4.2, Theorems 4.10 and 4.13, and Corollary 4.25). Our strategy is as follows: We begin with a pointwise bound of the Fourier transform. In order to improve on this result for small frequencies, we expand the Fourier transform in a Taylor series about the origin. For technical reasons, we consider the contributions of even and odd parity separately. We successively derive more and more refined estimates for the Taylor coefficients. In the final step, we prove several bounds for the Taylor series in closed form. Our estimates will be presented in increasing level of refinement and, accordingly, in increasing complexity of the proofs.

### A Pointwise Bound of the Fourier Transform

We begin with a simple and well-known pointwise bound for the Fourier transform. It will serve as a reference for the improved bounds for small frequencies to be derived later on. For our estimates, it is useful to introduce the functions

$$\begin{aligned} \hat{h}_\pm (k) := \omega \, \hat{\phi }_\pm (0,k) \end{aligned}$$

with $\omega$ as in (4.4), where for convenience we evaluated at time $t=0$. According to Lemma 4.1, the energy $E(\phi _\pm )$ simply is a multiple of the $L^2$-norm of $\hat{h}_\pm (k)$ squared. The following estimates apply similarly to both $\hat{h}_+$ and $\hat{h}_-$. We begin with a pointwise bound.

#### Lemma 4.2

For all $$k \in \mathbb {R}$$,

$$\begin{aligned} \big |\hat{h}_\pm (k)\big | \le \sqrt{2 E(\phi )}{.} \end{aligned}$$

#### Proof

According to (4.3),

$$\begin{aligned} \big |\hat{h}_\pm (k) \big | = |k\, \hat{\phi }_\pm (k)| \le \frac{1}{2} \, \Big ( |k\, \hat{\phi }_0(k)| + |\hat{\phi }_1(k)| \Big ) \le \frac{1}{\sqrt{2}}\, \Big ( |k\, \hat{\phi }_0(k)|^2 + |\hat{\phi }_1(k)|^2 \Big )^\frac{1}{2} {.} \end{aligned}$$

The obtained Fourier transforms can be estimated pointwise by

$$\begin{aligned} \big | k \,\hat{\phi }_0(k) \big |&\le \bigg | \int _{B_1} \partial _x\phi _0(x) \, e^{-i k x}\, \mathrm{d}x \bigg | \le \int _{B_1} \big |\partial _x\phi _0(x)\big | \, \mathrm{d}x \le \sqrt{2}\, \Vert \partial _x\phi _0\Vert _{L^2(B_1)} \\ \big | \hat{\phi }_1(k) \big |&\le \bigg | \int _{B_1} \phi _1(x) \, e^{-i k x}\, \mathrm{d}x \bigg | \le \int _{B_1} \big |\phi _1(x)\big | \, \mathrm{d}x \le \sqrt{2}\, \Vert \phi _1\Vert _{L^2(B_1)} {.} \end{aligned}$$

Comparing with (4.2) evaluated at time $t=0$ gives the result. $\square$

The goal of the following sections is to improve this estimate of $|\hat{h}_\pm (k)|$ for small *k*.

### Taylor Expansion in Momentum Space

Our first step is to expand the initial data $\hat{\phi }_{0/1}$ as well as the corresponding solutions $\phi _\pm$ of positive and negative frequency in Taylor series about the momentum $k=0$. Since the initial data is compactly supported, its Fourier transform is real analytic (for a proof of this statement see Lemma 2.1). Therefore, we may expand the initial data in Taylor series,

4.6 $$\begin{aligned} \hat{\phi }_0(k) = \sum _{n=0}^\infty \frac{\hat{\phi }^{(n)}_0(0)}{n!}\, k^n \qquad \text {and} \qquad \hat{\phi }_1(k) = \sum _{n=0}^\infty \frac{\hat{\phi }^{(n)}_1(0)}{n!}\, k^n {.} \end{aligned}$$

Using these formulas in (4.3), we obtain corresponding series expansions for the solutions $\hat{\phi }_\pm$ (we evaluate at $t=0$ and leave out the argument *t*),

$$\begin{aligned} \hat{\phi }_\pm (k) = \frac{1}{2} \, \Big ( \hat{\phi }_0(k) \pm \frac{i}{\omega }\, \hat{\phi }_1(k) \Big ) = \frac{1}{2} \sum _{n=0}^\infty \bigg ( \frac{\hat{\phi }^{(n)}_0(0)}{n!} \pm \frac{i}{\omega }\, \frac{\hat{\phi }^{(n)}_1(0)}{n!} \bigg )\, k^n {.} \end{aligned}$$

According to Lemma 4.1, the energy is the $L^2$-norm of $\omega \, \hat{\phi }_\pm (k)$. Therefore, we multiply by $\omega$. Using that $\omega =|k|$, we obtain

4.7 $$\begin{aligned} \hat{h}_\pm (k) = \omega \, \hat{\phi }_\pm (k)&= \frac{1}{2} \sum _{n=0}^\infty \bigg ( \omega \, \frac{\hat{\phi }^{(n)}_0(0)}{n!} \pm i\, \frac{\hat{\phi }^{(n)}_1(0)}{n!} \bigg )\, k^n \nonumber \\&= \frac{1}{2} \sum _{n=0}^\infty \bigg ( \epsilon (k)\, \frac{\hat{\phi }^{(n)}_0(0)}{n!} \, k^{n+1} \pm i\, \frac{\hat{\phi }^{(n)}_1(0)}{n!} \, k^n \bigg ) {,} \end{aligned}$$

where $\epsilon (k)$ is again the sign function. This sign function is crucial for what follows. Its significance becomes clear from the fact that it is responsible for Hegerfeldt’s theorem to hold: Assume that $\hat{\phi }_-$ vanishes. Then, the series in (4.7) must vanish for all $k \in \mathbb {R}$. Hence, the coefficient of every power in |*k*| must be zero, i.e.,

$$\begin{aligned} \hat{\phi }^{(0)}_0(0) = 0 \quad \text {and} \quad \epsilon (k)\, \frac{\hat{\phi }^{(n-1)}_0(0)}{(n-1)!}+ i\, \frac{\hat{\phi }^{(n)}_1(0)}{n!} = 0 \quad \text {for all } n \ge 1{.} \end{aligned}$$

This equation must hold for both signs of *k*, i.e.,

$$\begin{aligned} \frac{\hat{\phi }^{(n-1)}_0(0)}{(n-1)!}+ i\, \frac{\hat{\phi }^{(n)}_1(0)}{n!}&= 0 \qquad \text {for } k>0 \\ -\frac{\hat{\phi }^{(n-1)}_0(0)}{(n-1)!}+ i\, \frac{\hat{\phi }^{(n)}_1(0)}{n!}&= 0 \qquad \text {for } k<0{.} \end{aligned}$$

As a consequence, all the summands in (4.7) must be zero, implying that the initial data vanishes identically. This simple argument even makes it possible to quantify Hegerfeldt’s theorem. Indeed, if $\hat{\phi }_-$ is small, then all its Taylor coefficients are small, implying that also the initial data must be small. Clearly, our task is to specify what “small” means and to derive corresponding estimates.

In preparation of this analysis, we now express the energy of $\phi _\pm$ in terms of the initial data. It is useful to decompose the solution with respect to parity, i.e., the symmetry under spatial reflections at the origin. Thus, for a function $\phi (t,x)$ we introduce the parity decomposition by

$$\begin{aligned} \phi (t,x) = \phi ^\mathrm{even}(t,x) + \phi ^\mathrm{odd}(t,x) {,} \end{aligned}$$

where

$$\begin{aligned} \phi ^\mathrm{even}(t,x) := \frac{1}{2} \Big ( \phi (t,x) + \phi (t,-x) \Big ) \quad \text {and} \quad \phi ^\mathrm{odd}(t,x) := \frac{1}{2} \Big ( \phi (t,x) - \phi (t,-x) \Big ) {.} \end{aligned}$$

Since the Fourier transform preserves parity, we obtain similar decompositions in momentum space, namely

$$\begin{aligned} \hat{\phi }^\mathrm{even}(k) = \frac{1}{2} \Big ( \hat{\phi }(k) + \hat{\phi }(-k) \Big ) \qquad \text {and} \qquad \hat{\phi }^\mathrm{odd}(k) = \frac{1}{2} \Big ( \hat{\phi }(k) - \hat{\phi }(-k) \Big ) {.} \end{aligned}$$

Having fixed the parity, it clearly suffices to analyze $\hat{\phi }^{\mathrm{even}/\mathrm{odd}}$ for positive *k*, implying that $k=|k|=\omega$. Therefore, it is unnecessary to distinguish between *k* and $\omega$. Comparing with (4.7), we obtain

4.8 $$\begin{aligned} \hat{h}^\mathrm{even}_\pm (\omega ) = \sum _{n=0}^\infty a_n^\mathrm{even}\, \omega ^n \qquad \text {and} \qquad \hat{h}^\mathrm{odd}_\pm (\omega ) = \sum _{n=1}^\infty a_n^\mathrm{odd}\, \omega ^n {,} \end{aligned}$$

where the series coefficients of even and odd parity are given by

4.9 $$\begin{aligned} a^\mathrm{even}_{2\ell }&= \pm \frac{i}{2} \, \frac{\hat{\phi }_1^{(2\ell )}(0)}{(2\ell )!} {,}\quad&a^\mathrm{even}_{2\ell +1}&= \frac{1}{2}\, \frac{\hat{\phi }_0^{(2\ell )}(0)}{(2\ell )!} \end{aligned}$$

4.10 $$\begin{aligned} a^\mathrm{odd}_{2\ell +2}&= \frac{1}{2}\, \frac{\hat{\phi }_0^{(2\ell +1)}(0)}{(2\ell +1)!} {,}\quad&a^\mathrm{odd}_{2\ell +1}&= \pm \frac{i}{2}\, \frac{\hat{\phi }_1^{(2\ell +1)}(0)}{(2\ell +1)!} {.} \end{aligned}$$

#### Lemma 4.3

The energy of the positive- and negative-frequency components of $\phi$ as given in Lemma 4.1 can be written as

$$\begin{aligned} E(\phi _\pm ) = E(\phi ^\mathrm{even}_\pm ) + E(\phi ^\mathrm{odd}_\pm ) \end{aligned}$$

with

4.11 $$\begin{aligned} \begin{aligned} E\big (\phi ^\mathrm{even}_\pm \big )&= \frac{1}{\pi } \int _0^\infty \bigg | \sum _{n=0}^\infty a^\mathrm{even}_n\, \omega ^n \bigg |^2\, \mathrm{d}\omega \\ E\big (\phi ^\mathrm{odd}_\pm \big )&= \frac{1}{\pi } \int _0^\infty \bigg | \sum _{n=1}^\infty a^\mathrm{odd}_n\, \omega ^n \bigg |^2\, \mathrm{d}\omega {.} \end{aligned} \end{aligned}$$

#### Proof

Using (4.5), we obtain

$$\begin{aligned} E(\phi _\pm )&= \int _{-\infty }^\infty \frac{\mathrm{d}k}{2 \pi } \,\big | \hat{h}_\pm (k) \big |^2 = \int _0^\infty \frac{\mathrm{d}\omega }{2 \pi } \,\Big ( \big | \hat{h}_\pm (\omega ) \big |^2 + \big | \hat{h}_\pm (-\omega ) \big |^2 \Big ) \\&= \frac{1}{4 \pi } \int _0^\infty \Big ( \big | \hat{h}_\pm (\omega ) + \hat{h}_\pm (-\omega ) \big |^2 + \big | \hat{h}_\pm (\omega ) - \hat{h}_\pm (-\omega ) \big |^2 \Big ) \, \mathrm{d}\omega {.} \end{aligned}$$

The two summands in the integrand are the even and odd parity components, respectively. Computing them using (4.8) gives the result. $\square$

### Simple Estimates of the Taylor Coefficients

The following estimates apply to both series in (4.11) in the same way. For notational convenience, the superscript $\bullet$ stands for either “even” or “odd.” Thus, we write the series in (4.11) as

4.12 $$\begin{aligned} \hat{h}_\pm ^\bullet (\omega ) := \sum _{n=0}^\infty a_n^\bullet \,\omega ^n \,:\, \mathbb {R}^+ \rightarrow \mathbb {C}{,} \end{aligned}$$

where we set $a_0^\mathrm{odd}=0$. Our goal is to estimate the functions $\hat{h}_\pm ^\bullet (\omega )$ for low frequencies. Before entering this analysis, we point out that, according to (4.9) and (4.10), the coefficients $a_n^\bullet$ differ in the cases $+$ and − only by signs. Therefore, whenever we estimate the absolute values of these coefficients, the distinction between the cases $+$ and − becomes irrelevant. Moreover, from (4.9) and (4.10) one sees that the series involving the absolute values of the coefficients bounds the initial data in the sense that

$$\begin{aligned} 2\, \big | \hat{h}^\bullet _\pm (k) \big | \le \big | k\, \hat{\phi }^\bullet _0(k) \big | + \big | \hat{\phi }^\bullet _1(k) \big | \le \sum _{n=0}^\infty \big |a_n^\bullet \big | \,\omega ^n {.} \end{aligned}$$

These inequalities will be crucial for the following estimates.

We begin with a simple estimate of each coefficient of the series expansion, which is based on Lemma 2.1.

#### Proposition 4.4

The coefficients in the power series (4.12) are bounded by

$$\begin{aligned} |a^\bullet _n| \le \frac{\sqrt{E(\phi ^\bullet )}}{n!} {.} \end{aligned}$$

#### Proof

Using the result of Lemma 2.1 in (4.9) and (4.10), one finds that the coefficients $a^\bullet _n$ are bounded by

$$\begin{aligned} \big |a^\mathrm{even}_{2\ell } \big |&\le \frac{1}{\sqrt{2}}\, \frac{1}{(2\ell )!} \, \Vert \phi ^\mathrm{even}_1\Vert _{L^2(B_1)}{,}&\big |a^\mathrm{even}_{2\ell +1} \big |&\le \frac{1}{\sqrt{2}}\, \frac{1}{(2\ell +1)!} \, \Vert \partial _x \phi ^\mathrm{even}_0\Vert _{L^2(B_1)} \\ \big |b^\mathrm{odd}_{2\ell +2} \big |&\le \frac{1}{\sqrt{2}}\, \frac{1}{(2\ell +2)!} \, \Vert \partial _x \phi ^\mathrm{odd}_0\Vert _{L^2(B_1)}{,}&\big |b^\mathrm{odd}_{2\ell +1} \big |&\le \frac{1}{\sqrt{2}}\, \frac{1}{(2\ell +1)!} \, \Vert \phi ^\mathrm{odd}_1\Vert _{L^2(B_1)} {.} \end{aligned}$$

We thus obtain the simple bound in terms of the energy

$$\begin{aligned} |a^\bullet _n|&\le \frac{1}{n!}\, \frac{1}{\sqrt{2}}\, \max \Big \{ \Vert \partial _x\phi ^\bullet _0\Vert _{L^2(B_1)},\, \Vert \phi ^\bullet _1\Vert _{L^2(B_1)} \Big \} \\&\le \frac{1}{n!}\, \frac{1}{\sqrt{2}}\, \sqrt{ \Vert \partial _x\phi ^\bullet _0\Vert _{L^2(B_1)}^2 + \Vert \phi ^\bullet _1\Vert _{L^2(B_1)}^2 } = \frac{\sqrt{E(\phi ^\bullet )}}{n!} {.} \end{aligned}$$

This concludes the proof. $\square$

### Estimates of the Highest Coefficient of a Polynomial

In Proposition 4.4, the Taylor coefficients were estimated in terms of the total energy $E(\phi ^\bullet )$ of the wave. However, it was not taken into account that the corresponding Taylor series describes the component of positive or negative frequency only (see (4.8)). More specifically, we consider the situation when the energy of the negative-frequency component is much smaller than the total energy,

$$\begin{aligned} E(\phi ^\bullet _-) \ll E(\phi ^\bullet ) {.} \end{aligned}$$

Choosing the plus sign in (4.8), we are interested in upper bounds of the Taylor coefficients in (4.12), which tend to zero if $E(\phi ^\bullet _-)$ tends to zero for fixed $E(\phi ^\bullet )$. In order to derive these refined estimates, we use the following strategy, which is similar to that used by Tao to prove a version of Hardy’s uncertainty principle in [28,  Sect. 2.6.2., p. 360]. We decompose the Taylor series into a Taylor polynomial of degree *N* and the remainder term,

4.13 $$\begin{aligned} \hat{h}_\pm ^\bullet = \hat{h}^\bullet _N + R^\bullet _N \quad \text {with} \quad \hat{h}^\bullet _N(\omega ) := \sum _{n=0}^N a^\bullet _n \, \omega ^n {,}\quad R^\bullet _N(\omega ) := \sum _{n=N+1}^\infty a^\bullet _n \, \omega ^n {.}\nonumber \\ \end{aligned}$$

We first show that if the Taylor polynomial has small $L^2$-norm on an interval $[0, \omega _1]$, then its highest coefficient must also be small. This statement is quantified in the following lemma using properties of the Legendre polynomials. Combining this statement with an $L^2$-estimate of the remainder term (see Lemma 4.6 in the next section), we shall obtain the refined estimates of each Taylor coefficient in Proposition 4.7.

#### Lemma 4.5

Let ${\mathcal {P}}(\omega )$ be a real polynomial of degree at most *N* with $N \in \mathbb {N}_0$,

$$\begin{aligned} {\mathcal {P}}(\omega ) = a_0 + a_1\, \omega + \cdots + a_N\, \omega ^N {.} \end{aligned}$$

Then, for any $\omega _1>0$, the highest coefficient of ${\mathcal {P}}$ satisfies the following inequalities:4.144.15

#### Proof

For notational simplicity, we arrange by a rescaling that $\Vert {\mathcal {P}}\Vert _{L^2([0,\omega _1])} = 1$. We make use of the fact that the Legendre polynomials $P_n$ are orthogonal in $L^2([-1,1])$. More precisely, for all $n,n' \in \mathbb {N}_0$ (see [22,  Table 18.3.1])

$$\begin{aligned} \int _{-1}^1 P_n(x)\, P_{n'}(x) = \frac{2}{2n+1}\, \delta _{n,n'} {.} \end{aligned}$$

Combining this orthogonality with the fact that the Legendre polynomials $P_0, \ldots , P_{N-1}$ are a basis of the polynomials of degree at most $N-1$, we conclude that the Legendre polynomial $P_N$ is orthogonal to all polynomials of degree smaller than *N*. It follows that

$$\begin{aligned} \int _{0}^{\omega _1} {\mathcal {P}}(\omega )\, P_{N}\Big (\frac{2\omega }{\omega _1}- 1 \Big ) \,\mathrm{d}\omega = \int _0^{\omega _1} a_N \,\omega ^N\, P_{N}\Big (\frac{2\omega }{\omega _1}- 1 \Big )\,\mathrm{d}\omega {.} \end{aligned}$$

This makes it possible to compute the coefficient $a_N$ by

4.16 $$\begin{aligned} a_N = \frac{1}{c_N} \int _{0}^{\omega _1} {\mathcal {P}}(\omega )\, P_{N}\Big (\frac{2\omega }{\omega _1}- 1 \Big ) \,\mathrm{d}\omega \quad \text {with} \quad c_N := \int _0^{\omega _1} \omega ^N\, P_{N}\Big (\frac{2\omega }{\omega _1}- 1 \Big )\,\mathrm{d}\omega {.}\nonumber \\ \end{aligned}$$

The first integral can be estimated with the help of the Schwarz inequality by

4.17 $$\begin{aligned}&\bigg | \int _{0}^{\omega _1} {\mathcal {P}}(\omega )\, P_{N}\Big (\frac{2\omega }{\omega _1}- 1 \Big ) \,\mathrm{d}\omega \bigg | \le \Vert {\mathcal {P}}\Vert _{L^2([0,\omega _1], \mathrm{d}\omega )} \bigg ( \int _{0}^{\omega _1} \Big | P_{N}\Big (\frac{2\omega }{\omega _1}- 1 \Big ) \Big |^2\,\mathrm{d}\omega \bigg )^\frac{1}{2} \nonumber \\&\quad \le \sqrt{\frac{\omega _1}{2}} \,\Vert P_{N}\Vert _{L^2([-1,1])} = \sqrt{\frac{\omega _1}{2}} \, \frac{\sqrt{2}}{\sqrt{2N+1}} = \frac{\sqrt{\omega _1}}{\sqrt{2N+1}} {.} \end{aligned}$$

The second integral in (4.16), on the other hand, can be computed explicitly. First, introducing the integration variable $x=2\omega /\omega _1 - 1$, we find that

$$\begin{aligned} c_N&= \frac{\omega _1}{2} \int _{-1}^1 \Big ( \frac{\omega _1\, (x+1)}{2}\Big )^N\, P_{N}(x)\,\mathrm{d}x = \Big ( \frac{\omega _1}{2} \Big )^{N+1} \int _{-1}^1 (x+1)^N\, P_{N}(x)\,\mathrm{d}x \\&= \Big ( \frac{\omega _1}{2} \Big )^{N+1} \int _{-1}^1 x^N\, P_{N}(x)\,\mathrm{d}x = \Big ( \frac{\omega _1}{2} \Big )^{N+1} \,2 \int _0^1 x^N\, P_{N}(x)\,\mathrm{d}x{,} \end{aligned}$$

where in the last line we again used that $P_N$ is orthogonal to all polynomials of degree smaller than *N*. We now employ the relations (see [22,  Eqs. 18.17.38 and 18.17.39]) together with the Stirling formula (see [22,  Eq. 5.11.3 with leading term]),We thus obtain the estimateEmploying the above estimates in (4.16) gives (4.14).

Clearly, relation (4.14) implies that (4.15) holds for large *N*. In order to also verify (4.15) for small *N*, one can estimate the above combinatorial factors directly to obtain

$$\begin{aligned} \int _{0}^{1} P_{2n}\left( x\right) x^{2n}\mathrm {d}x&\ge \frac{1}{\sqrt{2\,(2n)+1}\, 2^{2n}} \\ \int _{0}^{1} P_{2n+1}\left( x\right) x^{2n+1}\mathrm {d}x&\ge \frac{1}{\sqrt{2\,(2n+1)+1}\, 2^{2n+1}} {.} \end{aligned}$$

As a consequence,

$$\begin{aligned} c_N \ge \Big ( \frac{\omega _1}{2} \Big )^{N+1}\, \frac{1}{\sqrt{N+1}\, 2^N} {.} \end{aligned}$$

Using this estimate together with (4.17) in (4.16) gives (4.15). $\square$

### Smallness of the Taylor Coefficients

We next estimate the $L^2$-norm of the remainder term in (4.13) on an interval $[0, \omega _1]$.

#### Lemma 4.6

Given $\varepsilon \in [0, 1]$ and $N \in \mathbb {N}_0$, we choose

4.18 $$\begin{aligned} \omega _1 = \Big ( \varepsilon ^2\, (N+1)!^2\, (2N+3) \Big )^{\frac{1}{2N+3}} {.} \end{aligned}$$

Then, the remainder term in (4.13) is bounded on $[0, \omega _1]$ by

$$\begin{aligned} \Vert R^\bullet _N(\omega )\Vert _{L^2([0, \omega _1])} \le 4 \varepsilon \; \sqrt{E(\phi ^\bullet )} {.} \end{aligned}$$

#### Proof

Applying Proposition 4.4, we can estimate the remainder by

4.19 $$\begin{aligned} |R^\bullet _N(\omega )|&\le \sum _{n=N+1}^\infty \frac{\omega ^n}{n!} \, \sqrt{E(\phi ^\bullet )} \nonumber \\&= \frac{\omega ^{N+1}}{(N+1)!} \,\Big ( 1 + \frac{\omega }{N+2} + \frac{\omega ^2}{(N+2)(N+3)} + \cdots \Big ) \, \sqrt{E(\phi ^\bullet )} \nonumber \\&\le c(\omega )\, \frac{\omega ^{N+1}}{(N+1)!} \,\sqrt{E(\phi ^\bullet )}\qquad \text {with} \qquad c(\omega ) := \sum _{n=0}^\infty \Big ( \frac{\omega }{N+2} \Big )^n {.} \end{aligned}$$

Choosing $\omega _1$ according to (4.18), we know that for all $\omega \in [0,\omega _1]$,

$$\begin{aligned} \frac{\omega }{N+2} \le \frac{\omega _1}{N+2} \le \frac{\big ( (N+1)!^2\, (2N+3) \big )^{\frac{1}{2N+3}}}{N+2} \le \frac{3}{4}{,} \end{aligned}$$

where the last inequality is verified by direct inspection and using the Stirling formula. Therefore, the geometric series in (4.19) converges and is bounded by four,

$$\begin{aligned} |R^\bullet _N(\omega )| \le 4\, \frac{\omega ^{N+1}}{(N+1)!}\, \sqrt{E(\phi ^\bullet )} {.} \end{aligned}$$

Using this pointwise bound, the $L^2$-norm can be estimated by

$$\begin{aligned} \Vert R^\bullet _N(\omega )\Vert ^2_{L^2([0, \omega _1]} \le 16\, E(\phi ^\bullet ) \int _0^{\omega _1} \frac{\omega ^{2N+2}}{(N+1)!^2}\, \mathrm{d}\omega \le \frac{16\, E(\phi ^\bullet )}{(N+1)!^2\, (2N+3)} \,\omega _1^{2N+3} {,} \end{aligned}$$

giving the result. $\square$

#### Proposition 4.7

Assume that

$$\begin{aligned} E(\phi ^\bullet _-) \le \varepsilon ^2\, E(\phi ^\bullet ) {.} \end{aligned}$$

Then, the Taylor coefficients in (4.12) are bounded for all $n \in \mathbb {N}_0$ by

$$\begin{aligned} |a^\bullet _n| \le \frac{6}{\sqrt{2n+1}}\, \frac{4^n}{n!}\, \varepsilon ^{\frac{2}{2n+3}} \, \sqrt{E(\phi ^\bullet )}{.} \end{aligned}$$

#### Proof

Given $N \in \mathbb {N}_0$, we choose $\omega _1$ as in (4.18). Then, the $L^2$-norm of the remainder is bounded according to Lemma 4.6. Combining this fact with Lemma 4.3, we obtain

$$\begin{aligned} \Vert \hat{h}^\bullet _N(\omega )\Vert _{L^2([0, \omega _1])}&= \big \Vert \hat{h}_\pm ^\bullet - R_N^\bullet \big \Vert _{L^2([0, \omega _1])} \le \big \Vert \hat{h}_\pm ^\bullet \big \Vert _{L^2([0, \omega _1])} + \big \Vert R_N^\bullet \big \Vert _{L^2([0, \omega _1])} \\&\le \big \Vert \hat{h}_\pm ^\bullet \big \Vert _{L^2([0, \infty ))} + \big \Vert R_N^\bullet \big \Vert _{L^2([0, \omega _1])}\\&\le \sqrt{\pi \, E(\phi ^\bullet _-)} + \Vert R_N^\bullet \Vert _{L^2([0, \omega _1])} \\&\le \varepsilon \, \sqrt{\pi \, E(\phi ^\bullet )} + 4 \varepsilon \; \sqrt{E(\phi ^\bullet )} \le 6 \varepsilon \, \sqrt{E(\phi ^\bullet )} {.} \end{aligned}$$

Applying Lemma 4.5 to the polynomial $\hat{h}^\bullet _N$ gives the bound

$$\begin{aligned} |a^\bullet _N|&\le \frac{1}{\sqrt{\omega _1}}\, \Big ( \frac{4}{\omega _1} \Big )^N\, 6 \varepsilon \, \sqrt{E(\phi ^\bullet )} \\&= \varepsilon ^{\frac{2}{2N+3}} \, 4^N\, (N+1)!^{-\frac{2N+1}{2N+3}}\, (2N+3)^{-\frac{2N+1}{4N+6}}\, 6\, \sqrt{E(\phi ^\bullet )} {.} \end{aligned}$$

The result follows asymptotically from the Stirling formula and for small values of *n* directly by numerical evaluation. $\square$

### Smallness of the Initial Data

In Proposition 4.7, we estimated all the Taylor coefficients $a^\bullet _n$. According to (4.9) and (4.10), this also gives control of all the Taylor coefficients of the initial data $\hat{\phi }_0$ and $\hat{\phi }_1$. We thus obtain the following result.

#### Proposition 4.8

Assume that the energy of the negative-frequency component is bounded in terms of the total energy by

$$\begin{aligned} E(\phi ^\bullet _-) \le \varepsilon ^2\, E(\phi ^\bullet ) {.} \end{aligned}$$

Then, the even and odd components of the initial data in momentum space are bounded pointwise for all $\omega \in \mathbb {R}^+$ by

$$\begin{aligned} 2\,\big |\hat{h}_\pm ^\bullet (\omega )\big |\le \big | \omega \,\hat{\phi }^\bullet _0(\omega ) \big | + \big | \hat{\phi }^\bullet _1(\omega ) \big | \le 12\, \sqrt{E(\phi ^\bullet )} \; \big ( 4\omega \big )^{-\frac{3}{2}}\, g\big (\omega , \varepsilon \big ) {,} \end{aligned}$$

where *g* is the series

4.20 $$\begin{aligned} g(\omega , \varepsilon ) := \sum _{n=0}^\infty \frac{1}{\sqrt{2n+1}}\, \frac{(4 \omega )^{n+\frac{3}{2}}}{n!}\, \varepsilon ^{\frac{2}{2n+3}} \end{aligned}$$

#### Proof

According to (4.6),

$$\begin{aligned} \big | k \,\hat{\phi }^\bullet _0(k) \big | + \big | \hat{\phi }^\bullet _1(k) \big | \le \sum _{n=0}^\infty \bigg ( \frac{|(\hat{\phi }^\bullet _0)^{(n)}(0)|}{n!}\, |k|^{n+1} + \frac{|(\hat{\phi }^\bullet _1)^{(n)}(0)|}{n!}\, |k|^n \bigg ) {.} \end{aligned}$$

Using (4.9) and (4.10), one verifies for both the even and odd components that

$$\begin{aligned} \big | k \,\hat{\phi }^\bullet _0(k) \big | + \big | \hat{\phi }^\bullet _1(k) \big | \le 2 \sum _{n=0}^\infty |a_n^\bullet |\, |k|^n{.} \end{aligned}$$

Applying the estimate of Proposition 4.7 gives the result. $\square$

Before studying series (4.20) in detail and deriving bounds in closed form, we explain how to derive corresponding estimates for both parity components together (i.e., without decomposing into even and odd components).

#### Theorem 4.9

Assume that the energy of the negative-frequency component is bounded in terms of the total energy by

$$\begin{aligned} E(\phi _-) \le \varepsilon ^2\, E(\phi ) {.} \end{aligned}$$

Then, we have the pointwise bound

$$\begin{aligned} \big |\hat{h}_\pm (k)\big |\le 12\,\sqrt{E(\phi )} \,(4\omega )^{-\frac{3}{2}}\,g(\omega ,\varepsilon ) {.} \end{aligned}$$

#### Proof

Clearly, we may assume that both $E(\phi ^{\mathrm{odd}})$ and $E(\phi ^{\mathrm{even}})$ are nonzero, because otherwise the result follows immediately from Proposition 4.8. Since *g* is monotone increasing in $\varepsilon$, we may assume that

4.21 $$\begin{aligned} E(\phi _-)=\varepsilon ^2\, E(\phi ) {.} \end{aligned}$$

Setting $\delta = E(\phi ^{\mathrm{odd}})/E(\phi ) \in (0,1)$ and using Lemmas 4.3 and 4.1, we find that

4.22 $$\begin{aligned} E(\phi ^{\mathrm{odd}}) = \delta \, E(\phi ) {,} \qquad E(\phi ^{\mathrm{even}}) = (1-\delta )\, E(\phi ) {.} \end{aligned}$$

Moreover, we introduce parameters $\varepsilon _\bullet \ge 0$ such that

4.23 $$\begin{aligned} E(\phi ^{\mathrm{odd}}_-) = \varepsilon _{\mathrm{odd}}^2\, E(\phi ^{\mathrm{odd}}) {,}\qquad E(\phi ^{\mathrm{even}}_-) = \varepsilon _{\mathrm{even}}^2\, E(\phi ^{\mathrm{even}}) {.} \end{aligned}$$

It follows that

$$\begin{aligned} \varepsilon ^2 E(\phi )&=E(\phi _-) =E(\phi ^{\mathrm{odd}}_-) + E(\phi ^{\mathrm{even}}_-) \nonumber \\&=\varepsilon ^2_{\mathrm{odd}}\,E(\phi ^{\mathrm{odd}})+ \varepsilon ^2_{\mathrm{even}}\,E(\phi ^{\mathrm{even}}) = \big (\varepsilon ^2_{\mathrm{odd}}\,\delta + \varepsilon ^2_{\mathrm{even}}\,(1-\delta ) \big )\, E(\phi ) {.} \end{aligned}$$

Solving for $\varepsilon _{\mathrm{even}}$ gives

$$\begin{aligned} \varepsilon _{\mathrm{even}}= \sqrt{\frac{\varepsilon ^2-\varepsilon _{\mathrm{odd}}^2\,\delta }{1-\delta }} {.} \end{aligned}$$

This relation shows that $\varepsilon _{\mathrm{odd}} \ge \varepsilon$ implies $\varepsilon _{\mathrm{even}} \le \varepsilon$ and vice versa. Therefore, we may assume without loss of generality that $\varepsilon _{\mathrm{even}} \le \varepsilon$ and $\varepsilon _{\mathrm{odd}}\ge \varepsilon$. (Otherwise, we repeat the following argument with odd and even components interchanged).

Next, it is straightforward to see that

$$\begin{aligned} |\hat{h}_\pm (k)|^2= & {} \big (|\hat{h}_\pm ^{\mathrm{odd}}(k)+ \hat{h}_\pm ^{\mathrm{even}}(k)| \big )^2 \le \big ( |\hat{h}_\pm ^{\mathrm{odd}}(k)|+ |\hat{h}_\pm ^{\mathrm{even}}(k)| \big )^2\\\le & {} 2\, \big (|\hat{h}_\pm ^{\mathrm{odd}}(k)|^2+ |\hat{h}_\pm ^{\mathrm{even}}(k)|^2 \big ) {.} \end{aligned}$$

Applying Proposition 4.8, we obtain

$$\begin{aligned} \big |\hat{h}_\pm (k)\big |^2\le \frac{288}{(4\omega )^3}\, \Big ( \delta \, g^2(\omega ,\varepsilon _{\mathrm{odd}}) + (1-\delta )\, g^2(\omega , \varepsilon _{\mathrm{even}}) \Big )\, E(\phi ) {.} \end{aligned}$$

Since *g* is monotone increasing in the argument $\varepsilon$, we may replace $\varepsilon _\mathrm{even}$ by $\varepsilon$. Moreover, combining (4.21) with (4.22) and (4.23), one sees that $\delta \le \varepsilon ^2/\varepsilon _{\mathrm{odd}}^2$. We thus obtain

4.24 $$\begin{aligned} \big |\hat{h}_\pm (k)\big |^2\le \frac{288}{(4\omega )^3}\left( g^2(\omega ,\varepsilon _{\mathrm{odd}}) \,\frac{\varepsilon ^2}{\varepsilon _{\mathrm{odd}}^2} + g^2\left( \omega ,\varepsilon \right) \right) E(\phi ). \end{aligned}$$

Finally, the computation

$$\begin{aligned}&\frac{\partial }{\partial \varepsilon _{\mathrm{odd}}} \bigg ( g^2(\omega ,\varepsilon _{\mathrm{odd}}) \,\frac{\varepsilon ^2}{\varepsilon _{\mathrm{odd}}^2} \bigg ) = \frac{ 2 \varepsilon ^2}{\varepsilon _{\mathrm{odd}}^3}\, g(\omega ,\varepsilon _{\mathrm{odd}}) \, \bigg (\varepsilon _{\mathrm{odd}} \,\frac{\partial g(\omega ,\varepsilon _{\mathrm{odd}})}{\partial \varepsilon _{\mathrm{odd}}}-g(\omega ,\varepsilon _{\mathrm{odd}})\bigg ) \\&\quad = \frac{ 2 \varepsilon ^2}{\varepsilon _{\mathrm{odd}}^3}\, g(\omega ,\varepsilon _{\mathrm{odd}}) \, \bigg ( \sum _{n=0}^\infty \frac{1}{\sqrt{2n+1}}\, \frac{(4 \omega )^{n+\frac{3}{2}}}{n!}\, \varepsilon _{\mathrm{odd}}^{\frac{2}{2n+3}} \,\Big (\frac{2}{2n+3}-1\Big ) \bigg ) <0 \end{aligned}$$

allows us to set $\varepsilon _{\mathrm{odd}}=\varepsilon$ in (4.24). This gives the result. $\square$

### A First Version of the Main Theorem

The remaining task is to estimate the series $g(\omega , \varepsilon )$ in (4.20), which we also write as

4.25 $$\begin{aligned} R(\omega , \varepsilon ) := (4\omega )^{-\frac{3}{2}} \,g(\omega , \varepsilon )= \sum _{n=0}^\infty \frac{1}{\sqrt{2n+1}}\, \frac{(4 \omega )^{n}}{n!}\, \varepsilon ^{\frac{2}{2n+3}} \end{aligned}$$

We now prove the first version of our main result.

#### Theorem 4.10

Assume that the energy of the negative-frequency component is bounded in terms of the total energy by

$$\begin{aligned} E(\phi ^\bullet _-) < \varepsilon ^2\, E(\phi ^\bullet ) {.} \end{aligned}$$

Then, the even and odd components of the initial data in momentum space are bounded pointwise for all $k \in \mathbb {R}$ by

4.26 $$\begin{aligned} 2\, \big |\hat{h}_\pm ^\bullet (k)\big |\le \big | k \,\hat{\phi }^\bullet _0(k) \big | + \big | \hat{\phi }^\bullet _1(k) \big | \le 6^{\frac{3}{2}}\, \frac{\sqrt{E(\phi ^\bullet )}}{\sqrt{2e\,|\log \varepsilon |}} \,e^{4\omega } {.} \end{aligned}$$

#### Proof

We estimate the series in (4.25) by

$$\begin{aligned}&\sum _{n=0}^\infty \frac{1}{\sqrt{2n+1}}\, \frac{(4 \omega )^{n}}{n!}\, \varepsilon ^{\frac{2}{2n+3}} \le \sqrt{\frac{3}{2}} \,\sum _{n=0}^\infty \sqrt{\frac{2}{2n+3}}\, \frac{(4 \omega )^{n}}{n!}\, \varepsilon ^{\frac{2}{2n+3}} \\&\le \sqrt{\frac{3}{2}} \max _{n\in [0,\infty )} \bigg [\sqrt{\frac{2}{2n+3}}\,\varepsilon ^{\frac{2}{2n+3}} \bigg ] \;\sum _{n=0}^\infty \frac{(4 \omega )^{n}}{n!} \le \sqrt{\frac{3}{2}} \, \sup _{x \in \mathbb {R}^+} \Big [ x\, e^{x^2 \log \varepsilon } \Big ] \,e^{4\omega }{,} \end{aligned}$$

where in the last step we set $x=\sqrt{2/(2n+3)}$. In order to estimate the last supremum, we set $y = \sqrt{-\log \varepsilon } x$,

$$\begin{aligned} \sup _{x \in \mathbb {R}^+} \Big [ x\, e^{x^2 \log \varepsilon } \Big ] = \frac{1}{\sqrt{-\log \varepsilon }}\, \sup _{y \in \mathbb {R}^+} y\, e^{-y^2} = \frac{1}{\sqrt{2 e \,|\log \varepsilon |}} {,} \end{aligned}$$

where we used that the function $y e^{-y^2}$ attains its maximum at $y=\sqrt{2}$. Combining this estimate with the result from Proposition 4.8 gives the result. $\square$

Note that the above estimate is an improvement over Lemma 4.2 as long as

$$\begin{aligned} \frac{6^{\frac{3}{2}}\,e^{4\omega }}{\sqrt{4e \,|\log \varepsilon |}}\le 1 {.} \end{aligned}$$

A straightforward calculation gives the following corollary:

#### Corollary 4.11

Assume that the energy of the negative-frequency component is bounded in terms of the total energy by

$$\begin{aligned} E(\phi ^\bullet _-) \le \varepsilon ^2\, E(\phi ^\bullet ) {.} \end{aligned}$$

Then, the $L^1$- and $L^2$-norms of the even and odd components of the initial data are bounded in momentum space for small frequencies

4.27 $$\begin{aligned} \omega \le \omega _{\max }(\varepsilon ) := \frac{1}{4}\, \log \left( \frac{\sqrt{2e\,|\log \varepsilon |}}{6^{\frac{3}{2}}}\right) \end{aligned}$$

by

$$\begin{aligned} \big \Vert \hat{h}_\pm ^\bullet (k)\big \Vert _{L^1([0,\omega _{\max }(\varepsilon )])} \le \frac{1}{8} \,\sqrt{E(\phi ^\bullet )} \quad \text { and } \quad \big \Vert \hat{h}_\pm ^\bullet (k)\big \Vert ^2_{L^2([0,\omega _{\max }(\varepsilon )])} \le \frac{1}{32} \,E(\phi ^\bullet ) {.} \end{aligned}$$

From Lemma 4.1, we know that the $L^2$-norm of $\hat{h}_\pm ^\bullet$ on the whole interval $[0, \infty )$ gives a multiple of the total energy. We thus obtain

$$\begin{aligned} \sum _{\pm } \int _{\omega _{\max }(\varepsilon )}^\infty \frac{\mathrm{d}k}{2 \pi }\, \omega ^2\,\big |\phi ^\bullet _\pm (k) \big |^2 \ge \Big ( 1 - \frac{1}{32\, \pi } \Big ) \, E(\phi ^\bullet ) {.} \end{aligned}$$

This inequality quantifies that the wave must have a significant high-energy contribution. Even more, as the function $\omega _{\max }(\varepsilon )$ is monotone decreasing in $\varepsilon \in (0,1]$ and tends to infinity as $\varepsilon \searrow 0$, we see that in this limiting case, the wave must have large contributions of higher and higher frequency.

We now give a less quantitative version of this result, which might be interesting in the context of a Littlewood–Paley decomposition.

#### Corollary 4.12

For every compact frequency range $[\omega _0,\omega _1] \subset \mathbb {R}$, every time $t_0 \in \mathbb {R}$ and every radius *r*, there is a constant $C<1$ such that the a priori estimate

$$\begin{aligned} E(\pi _{[\omega _0,\omega _1]}\phi ) \le C E(\phi ) \end{aligned}$$

holds for every smooth solution to the $1+1$-dimensional wave equation with

$$\begin{aligned} {{\,\mathrm{supp}\,}}\phi (t_0,.) \subset B_r {.} \end{aligned}$$

Here, $\pi _{[\omega _0,\omega _1]}\phi$ is the projection of the solution onto the compact frequency range.

#### Proof

By making the interval larger and arguing for positive and negative frequencies separately, it suffices to consider the case $\omega _0=0$ and $\omega _1>0$. Then, by choosing *C* sufficiently close to one, we can arrange that $\omega <\omega _{\max }$ with $\omega _{\max }$ as in (4.27) with $\varepsilon ^2 = 1-C$. Then, Corollary 4.11 gives the result.

$$\square $$

We presented a first straightforward estimate of the series and showed that it already allows us to derive interesting conclusions on the properties of solutions to the $1+1$-dimensional wave equation in the regime $E(\phi _-) \ll E(\phi )$. In the following, we will demonstrate that the bound on the series $g(\omega , \varepsilon )$ can be improved substantially. The conclusion on the qualitative level, however, will remain the same. Therefore, these improvements of the bounds are addressed more to technically-oriented readers.

### A First Improvement of the Estimate

In this section, we give a first improvement of the estimate in Theorem 4.10 by performing a more careful analysis of series (4.25). These estimates are a preparation for the more advanced method for getting estimates, which will be introduced in Sect. 4.9.

For ease in notation we set

4.28 $$\begin{aligned} a(\omega ) = \frac{\log (4 \omega )}{2} \qquad \text {and} \qquad b(\varepsilon ) = 2\,|\log \varepsilon | {.} \end{aligned}$$

Then, series (4.20) can be written as

4.29 $$\begin{aligned} g(a,b) := \sum _{n=0}^\infty \frac{1}{n!}\, \frac{1}{\sqrt{2n+1}}\,e^{(2n+3)\, a - \frac{b}{2n+3}} {.} \end{aligned}$$

Note that the last series converges absolutely and defines *g* as a smooth function on $\mathbb {R}^2$.

Here is the main result of this section:

#### Theorem 4.13

Let $\phi$ be the solution of the Cauchy problem (4.1). Assume that

$$\begin{aligned} E(\phi _-^\bullet ) \le \, \varepsilon ^2 \, E(\phi ^\bullet ) {.} \end{aligned}$$

Then, the initial data is small for small momenta in the sense that for all $\omega \ge 0$,

4.30 $$\begin{aligned} 2\,\big | \hat{h}_\pm ^\bullet (\omega )\big |\le & {} \big |\omega \hat{\phi }^\bullet _0(\pm \omega ) \big | +\; \big |\hat{\phi }^\bullet _1(\pm \omega ) \big | \nonumber \\\le & {} 12\, e^{4 \omega } \, \sqrt{E(\phi )} \,\max \bigg \{ \exp \Big ( -\frac{1}{14}\, \frac{|\log \varepsilon |}{\sqrt{\omega }} \Big ),\, e\, \exp \Big (- \sqrt{|\log \varepsilon |} \,\Big ) \bigg \} {.}\nonumber \\ \end{aligned}$$

#### Proof

In view of Proposition 4.8 and (4.28), (4.29), our task is to prove the following estimate,

$$\begin{aligned} g(a,b) \le 2\, e^{3a}\, \exp \big ( e^{2a} \big ) \,\max \bigg \{ \exp \Big ( -\frac{b}{14}\, e^{-a} \Big ),\, \exp \bigg (1 - \sqrt{\frac{b}{2}} \,\bigg ) \bigg \} {.} \end{aligned}$$

We begin with series (4.29), leaving out the factor $1/\sqrt{2n+1}$,

$$\begin{aligned} g(a,b) \le \sum _{n=0}^\infty \frac{1}{n!}\, e^{(2n+3)\,a - \frac{b}{2n+3}} {.} \end{aligned}$$

We decompose this series into the sum over the first *N* summands and the remainder. Estimating these two parts separately, we obtain

$$\begin{aligned} g(a,b)&\le \sum _{n=0}^N \frac{1}{n!}\, e^{(2n+3)\,a - \frac{b}{2n+3}} + \sum _{n=N+1}^\infty \frac{1}{n!}\, e^{(2n+3)\,a - \frac{b}{2n+3}} \\&\le e^{-\frac{b}{2N+3}} \sum _{n=0}^N \frac{1}{n!}\, e^{(2n+3)\,a} + \sum _{p=1}^\infty \frac{1}{(p+N)!}\, e^{(2p+2N+3)\,a - \frac{b}{2p+2N+3}} \\&\le e^{-\frac{b}{2N+3}} \, e^{3a}\, \exp \big ( e^{2a} \big ) \\&\quad + e^{-\frac{b}{2N+3}} \,\frac{e^{(2N+3)\,a}}{N!} \sum _{p=1}^\infty \frac{N!}{(p+N)!}\, e^{2pa - \frac{b}{2p+2N+3} + \frac{b}{2N+3}} \\&\overset{(*)}{\le } e^{-\frac{b}{2N+3}} \, e^{3a}\, \exp \big ( e^{2a} \big ) \bigg [ 1 + \sum _{p=1}^\infty \frac{N!}{(p+N)!}\, e^{2pa + \frac{2bp}{(2p+2N+3)(2N+3)}} \bigg ] \\&\le e^{-\frac{b}{2N+3}} \, e^{3a}\, \exp \big ( e^{2a} \big ) \bigg [ 1 + \sum _{p=1}^\infty \bigg ( \frac{1}{N+1}\, e^{2a + \frac{2b}{(2N+3)^2}} \bigg )^p \bigg ] {,} \end{aligned}$$

where in $(*)$ we used that

$$\begin{aligned} \frac{e^{2Na}}{N!} \le \sum _{n=0}^\infty \frac{e^{2na}}{n!} = \exp \big ( e^{2a} \big ) {.} \end{aligned}$$

Choosing *N* so large that

4.31 $$\begin{aligned} \frac{1}{N+1}\, e^{2a + \frac{2b}{(2N+3)^2}} \le \frac{1}{2}{,} \end{aligned}$$

we can compute the geometric series to obtain the estimate

$$\begin{aligned} g(a,b) \le 2\, e^{-\frac{b}{2N+3}} \, e^{3a}\, \exp \big ( e^{2a} \big ) {.} \end{aligned}$$

In order to satisfy condition (4.31), we first choose

$$\begin{aligned} 2N+3 \ge \,\sqrt{2 b} {,} \end{aligned}$$

which gives rise to the inequality

$$\begin{aligned} e^{\frac{2b}{(2N+3)^2}} \le e {.} \end{aligned}$$

Moreover, choosing

$$\begin{aligned} N+1 \ge 2\, e^{2a + 1} {,} \end{aligned}$$

we conclude that

$$\begin{aligned} \frac{1}{N+1}\, e^{2a + \frac{b}{(2N+3)^2}} \le \frac{1}{N+1}\, e^{2a + 1} \le \frac{1}{2} {,} \end{aligned}$$

implying that (4.31) holds. This leads us to choosing *N* as the integer in the range

$$\begin{aligned} N < \max \left\{ 2\, e^{2a+1}, \sqrt{\frac{b}{2}}-\frac{1}{2} \right\} \le N+1 {.} \end{aligned}$$

We thus obtain the estimates

$$\begin{aligned} 2N+3&\le \max \Big \{ 4\, e^{2a+1}+3, \sqrt{2b}+2 \Big \} \\ g(a,b)&\le 2\, e^{3a}\, \exp \big ( e^{2a} \big ) \,e^{-\frac{b}{2N+3}} \\&\le 2\, e^{3a}\, \exp \big ( e^{2a} \big ) \,\exp \bigg ( -\frac{b}{\max \big \{ 4\, e^{a+1}+3, \sqrt{2b} +2 \big \} } \bigg ) \\&= 2\, e^{3a}\, \exp \big ( e^{2a} \big ) \,\max \bigg \{ \exp \Big ( -\frac{b}{4\, e^{a+1}+3} \Big ),\, \exp \Big ( -\frac{b}{\sqrt{2b}+2} \Big ) \bigg \} {.} \end{aligned}$$

Employing the inequalities

$$\begin{aligned} \frac{1}{4\, e^{a+1}+3} \ge \frac{1}{14}\, e^{-a} \qquad \text {and} \qquad \frac{b}{\sqrt{2b}+2} \ge \sqrt{\frac{b}{2}} -1 \end{aligned}$$

gives the result. $\square$

We conclude this section with a comment on the parameter domains where the different estimates are better. We first evaluate the point where the two arguments of the maximum coincide. For simplicity disregarding the prefactor *e*, we obtain

$$\begin{aligned} \frac{1}{14}\, \frac{|\log \varepsilon |}{\sqrt{\omega }} = \sqrt{|\log \varepsilon |} \quad \Longleftrightarrow \quad \omega = \frac{|\log \varepsilon |}{196} {.} \end{aligned}$$

We thus obtain the estimate

$$\begin{aligned} \big | \hat{h}_\pm ^\bullet (\omega )\big | \le 24\,e\, e^{4 \omega } \, \sqrt{E(\phi )} \left\{ \begin{array}{cl} \displaystyle \exp \Big ( -\frac{1}{14}\, \frac{|\log \varepsilon |}{\sqrt{\omega }} \Big ) &{} \text {if } \displaystyle \omega > \frac{|\log \varepsilon |}{196} \\ \displaystyle \exp \Big (- \sqrt{|\log \varepsilon |} \,\Big ) &{} \text {if } \displaystyle \omega \le \frac{|\log \varepsilon |}{196} {.} \end{array} \right. \end{aligned}$$

For any given $\omega$, one finds that $|\hat{h}_\pm ^\bullet (\omega )| \lesssim \exp (-\sqrt{|\log \varepsilon |})$ asymptotically as $\varepsilon \searrow 0$. This is a faster decay than the asymptotics $|\hat{h}_\pm ^\bullet (\omega )| \lesssim 1/\sqrt{|\log \varepsilon |}$ as obtained in Theorem 4.10. On the other hand, fixing $\varepsilon$ and considering the asymptotics $\omega \rightarrow \infty$, the estimate of Theorem 4.10 is slightly better than that of Theorem 4.13 because of the factor $|\log \varepsilon |^{-\frac{1}{2}}$ in (4.26). However, in this limiting regime, both theorems are not useful, because the estimates are worse than the simple pointwise bound of Lemma 4.2. With this in mind, the above theorems are useful only for $\omega$ in a finite interval and for small $\varepsilon$.

We now turn to substantially more sophisticated techniques to obtain the best estimate in this paper (see Corollary 4.25).

### Formulation as a Goursat Problem for the Klein–Gordon Equation

We now develop another method for estimating the series *g* in (4.20). This method is based on the observation that *g* is a solution of a partial differential equation in $\varepsilon$ and $\omega$. As we shall see, this PDE is indeed the Klein–Gordon equation (see (4.32)), and the above series is obtained as the solution of a characteristic initial value problem (usually referred to as Goursat problem; see Proposition 4.14 below). This observation makes it possible to analyze the series in (4.20) with familiar methods of hyperbolic PDEs, as will be worked out in Sects. 4.11–4.12. Before entering the constructions, we remark that there seems no direct relation between the original wave equation and the PDE in $\varepsilon$ and $\omega$. To our knowledge, it is not even clear why *g* satisfies a PDE, and why this PDE is hyperbolic.

We again work with the parameters *a* and *b* as introduced in (4.28). Differentiating the function *g*(*a*, *b*) in (4.29) with respect to *a* and *b* gives

$$\begin{aligned} \partial _a g(a,b)&= \sum _{n=0}^\infty \frac{1}{n!}\, \frac{1}{\sqrt{2n+1}}\,(2n+3)\, e^{(2n+3)\, a - \frac{b}{2n+3}} \\ \partial _b \partial _a g(a,b)&= \sum _{n=0}^\infty \frac{1}{n!}\, \frac{1}{\sqrt{2n+1}}\, \Big (-\frac{2n+3}{2n+3} \Big )\, e^{(2n+3)\, a - \frac{b}{2n+3}} = -g(a,b){.} \end{aligned}$$

Hence, *g* is a solution of the PDE

4.32 $$\begin{aligned} (\partial _a \partial _b + 1)\, g = 0 {.} \end{aligned}$$

This is the $(1+1)$-dimensional Klein–Gordon equation of mass one in light cone coordinates. Introducing the coordinates

$$\begin{aligned} T&= a+b {,}&X&=a-b \\ \partial _T&= \frac{1}{2} \big ( \partial _a + \partial _b \big ) {,}&\partial _X&= \frac{1}{2} \big ( \partial _a - \partial _b \big ) {,} \end{aligned}$$

the equation takes the more familiar form

$$\begin{aligned} \big (\partial _T^2 - \partial _X^2 + 1 \big ) \,g = 0 {.} \end{aligned}$$

This PDE comes with initial conditions at $b=0$ given by the series

4.33 $$\begin{aligned} g_0(a) := g(a,0) = \sum _{n=0}^\infty \frac{1}{n!}\, \frac{1}{\sqrt{2n+1}}\,e^{(2n+3)\, a} {.} \end{aligned}$$

Moreover, Lebesgue’s monotone convergence theorem implies that

4.34 $$\begin{aligned} \lim _{b \rightarrow \infty } g(a,b) = \lim _{a \rightarrow -\infty } g(a,b) = 0 {.} \end{aligned}$$

The above PDE and the initial conditions determine the function *g* uniquely:

#### Proposition 4.14

The Goursat problem

4.35 $$\begin{aligned} \big ( \partial _a \partial _b + 1 \big )\,g(a,b) = 0 {,}\qquad g(a,0) = g_0(a) \end{aligned}$$

together with the decay conditions (4.34) has a unique solution in the half space

$$\begin{aligned} (a,b) \in \mathbb {R}\times \mathbb {R}^+_0 {.} \end{aligned}$$

It has the integral representation

4.36 $$\begin{aligned} g(a,b) = \int _{-\infty }^a J_0\Big ( 2\,\sqrt{(a-\tau ) \,b} \,\Big )\, g_0'(\tau )\, \mathrm{d}\tau {.} \end{aligned}$$

#### Proof

The appearance of the Bessel function in (4.36) can be understood directly from the form of the Green’s kernels of the Klein–Gordon equation as given in (2.8) and (2.9). Indeed, choosing the spacetime coordinates (*T*, *X*) and setting the mass to one, the causal fundamental solution (2.10) takes the form

$$\begin{aligned} K_1(T,X) = -\frac{i}{4 \pi } \, \epsilon (T) \,\Theta \big (T^2-X^2 \big ) \, J_0 \Big ( \sqrt{T^2-X^2} \Big ) \end{aligned}$$

where $\epsilon$ is again the sign function. Hence, in light cone coordinates,

4.37 $$\begin{aligned} K_1(T,X) = K[a,b] := -\frac{i}{4 \pi } \, \Theta (ab)\, \epsilon (b)\, J_0\Big ( 2\, \sqrt{ab} \Big ) \end{aligned}$$

(note that $T^2-X^2=(a+b)^2 - (a-b)^2 = 4 ab$). It is a solution of the homogeneous Klein–Gordon equation. Hence, also the convolution integral

$$\begin{aligned} h(a,b) := 4 \pi i \int _{-\infty }^\infty K[a-\tau , b]\, g_0'(\tau )\, \mathrm{d}\tau \end{aligned}$$

satisfies the Klein–Gordon equation. Using the explicit form of $K_1$ in (4.37), one sees that the function *h* coincides with the function *g* in (4.36).

Let us verify that the function *h* has the desired boundary values at $b=0$. Using that $J_0(0)=1$, we obtain

$$\begin{aligned} \lim _{b \searrow 0} h(a,b)&= \lim _{b \searrow 0} \int _{-\infty }^a J_0\Big ( 2\, \sqrt{(a-\tau )\,b}\, \Big )\, g_0'(\tau )\, \mathrm{d}\tau \\&= \int _{-\infty }^a g_0'(\tau )\, \mathrm{d}\tau = g_0(a){,} \end{aligned}$$

where we made use of the fact that $g_0(\tau )$ vanishes as $\tau \rightarrow -\infty$.

It remains to show uniqueness. Let $\tilde{g}$ be another solution of the Klein–Gordon equation with the same boundary values at $b=0$. Then, the difference $\phi :=g-\tilde{g}$ is a solution which vanishes at $b=0$. Our task is to prove that $\phi$ vanishes identically. This result can be understood intuitively from the fact that, being massive, a Klein–Gordon wave propagates with subluminal speed, implying that if it were nonzero, it would intersect the null line $b=0$. In order to prove this result, we consider the Fourier representation of $\phi$,

$$\begin{aligned} \phi (T,X) = \int _{-\infty }^\infty \Big ( \hat{\phi }_+(k)\, e^{-i \omega (k)\,T} + \hat{\phi }_-(k)\, e^{i \omega (k)\,T} \Big ) \,e^{ikX} {,} \end{aligned}$$

where $\omega (k):= \sqrt{k^1+1}$. The fact that $\phi$ vanishes on the line $b=0$ implies that

$$\begin{aligned} 0 = \phi (a,a) = \int _{-\infty }^\infty \Big ( \hat{\phi }_+(k)\, e^{-i \omega (k)\,a} + \hat{\phi }_-(k)\, e^{i \omega (k)\,a} \Big ) \,e^{ika} {.} \end{aligned}$$

Multiplying by $e^{i p a}$ and integrating over *a*, we obtain zero for any value of *p*. Since the mappings

$$\begin{aligned} \mathbb {R}\mapsto \mathbb {R}^\pm {,}\qquad k \mapsto k \pm \omega (k) \end{aligned}$$

are both injective, it follows that the functions $\hat{\phi }_\pm$ are both zero. Hence, $\phi$ vanishes identically. $\square$

We remark that identity (4.36) can also be derived without referring to hyperbolic PDEs simply by manipulating the power series; for details, see Appendix A.

### Arranging Initial Data in Closed Form

The initial data as given by series (4.33) has the disadvantage that it is not a simple explicit function. In view of the fact that the integral representation (4.36) involves the derivative of $g_0$ and that the Bessel function has an oscillatory behavior, it is not obvious how an estimate of the initial data translates into a corresponding estimate of the solution. For this reason, it is preferable to estimate the solution in terms of new solutions of the Goursat problem (4.35) for initial data given in closed form.

#### Lemma 4.15

The solution of the Goursat problem (4.35) with initial data (4.33) satisfies the inequality

$$\begin{aligned} \big | g(a,b) \big | \le \sqrt{ g^{(1)}(a,b) \, g^{(2)}(a,b) } {,} \end{aligned}$$

where the functions $g^{(1)}$ and $g^{(2)}$ are solutions of the Goursat problem (4.35) corresponding to the initial data

4.38 $$\begin{aligned} g_0^{(1)}(a) = e^{3a} \,\exp \big ( e^{2a} \big ) \qquad \text {and} \qquad g_0^{(2)}(a) = e^{3a} \int _0^1 \exp \big ( s^2 \,e^{2a} \big )\, \mathrm{d}s {,} \end{aligned}$$

respectively.

#### Proof

Since all summands in series (4.29) are non-negative, the Schwarz inequality gives

$$\begin{aligned} g(a,b)&= \sum _{n=0}^\infty \bigg ( \frac{1}{n!}\, e^{(2n+3)\, a - \frac{b}{2n+3}} \bigg )^\frac{1}{2} \; \bigg ( \frac{1}{n!}\, \frac{1}{2n+1}\,e^{(2n+3)\, a - \frac{b}{2n+3}} \bigg )^\frac{1}{2} \\&\le \bigg ( \sum _{n=0}^\infty \frac{1}{n!}\, e^{(2n+3)\, a - \frac{b}{2n+3}} \bigg )^\frac{1}{2} \bigg ( \sum _{n=0}^\infty \frac{1}{n!}\, \frac{1}{2n+1} \,e^{(2n+3)\, a - \frac{b}{2n+3}} \bigg )^\frac{1}{2} {.} \end{aligned}$$

By direct inspection, one sees that each bracket is a solution of the Goursat problem (4.35) corresponding to the initial data

$$\begin{aligned} g_0^{(1)}(a)&= \sum _{n=0}^\infty \frac{1}{n!}\, e^{(2n+3)\, a} = e^{3a} \,\sum _{n=0}^\infty \frac{1}{n!}\, \big ( e^{2a} \big )^n = e^{3a} \,\exp \big ( e^{2a} \big ) \qquad \text {and} \\ g_0^{(2)}(a)&= \sum _{n=0}^\infty \frac{1}{n!}\, \frac{1}{2n+1} \,e^{(2n+3)\, a} = e^{3a} \,\sum _{n=0}^\infty \frac{1}{n!}\, \frac{1}{2n+1} \,\big ( e^a \big )^{2n} \\&= e^{3a} \int _0^1 \bigg ( \sum _{n=0}^\infty \frac{1}{n!}\, s^{2n} \,\big ( e^a \big )^{2n} \bigg )\, \mathrm{d}s = e^{3a} \int _0^1 \exp \big ( s^2 \,e^{2a} \big )\, \mathrm{d}s {,} \end{aligned}$$

respectively. This concludes the proof. $\square$

### Reformulation as a Contour Integral

In this section, we rewrite the integral representation (4.36) in Proposition 4.14 as a contour integral. We make use of the fact that the Bessel function in (4.36) also arises in the causal fundamental solution (4.37), which in turn can be represented in momentum space by a distribution supported on the mass shell. Our starting point is formula (4.36). Introducing the integration variable

$$\begin{aligned} q := 2\,\sqrt{(a-\tau ) \,b} {,} \end{aligned}$$

we obtain

$$\begin{aligned} a-\tau = \frac{q^2}{4b} {,}\qquad \mathrm{d}\tau = \frac{1}{2b}\, q\, \mathrm{d}q \end{aligned}$$

and thus

$$\begin{aligned} g(a,b) = \frac{1}{2b} \int _0^\infty J_0(q)\, g_0'\Big ( a- \frac{q^2}{4b} \Big )\, q\, \mathrm{d}q {.} \end{aligned}$$

Since both functions $J_0$ and $g_0'$ are even in *t*, we can write this integral as

4.39 $$\begin{aligned} g(a,b) = \frac{1}{4b} \int _{-\infty }^\infty \Big ( J_0(q)\, \epsilon (q) \Big ) \, \Big ( g_0'\Big ( a- \frac{q^2}{4b} \Big )\, q \Big )\, \mathrm{d}q {.} \end{aligned}$$

Using Plancherel’s theorem, we can also compute this inner product in momentum space. In preparation, we compute the Fourier transform of the Bessel function:

#### Lemma 4.16

For any $$p \in \mathbb {R}$$,

$$\begin{aligned} \int _{-\infty }^\infty J_0(q)\, \epsilon (q)\, e^{i p q}\, \mathrm{d}q = 2 i \,\frac{\epsilon (p)}{\sqrt{p^2-1}}\, \chi _{\mathbb {R}\setminus [-1,1]}(p) \end{aligned}$$

where $\chi$ denotes the characteristic function and $\epsilon$ is again the sign function.

#### Proof

According to (4.37) and (2.12), for any $q \in \mathbb {R}$,

$$\begin{aligned} J_0(q)\, \epsilon (q)&= 4 \pi i\, K_1 \big (T=q,X=0\big ) = 4 \pi i \int \frac{\mathrm{d}\omega \, \mathrm{d}k}{(2 \pi )^2}\, \delta \big ( \omega ^2 - k^2-1\big )\, \epsilon (\omega )\, e^{-i \omega q} \\&= \frac{i}{\pi } \int _{-\infty }^\infty \mathrm{d}\omega \, \epsilon (\omega )\, e^{-i \omega q} \int _{-\infty }^\infty \delta \big (\omega ^2-k^2-1 \big )\, \mathrm{d}k \\&= \frac{i}{\pi } \int _{\mathbb {R}\setminus [-1,1]} \frac{\epsilon (\omega )}{\sqrt{\omega ^2-1}}\, e^{-i \omega q}\, \mathrm{d}\omega {.} \end{aligned}$$

We now apply Plancherel’s theorem. $\square$

#### Proposition 4.17

The function *g*(*a*, *b*) in (4.39) can be written as

4.40 $$\begin{aligned} g(a,b) = \frac{1}{\pi } \int _{\sqrt{2b}}^\infty \frac{k}{\sqrt{k^2-2b}}\, \hat{g}(a, k) \, \mathrm{d}k \end{aligned}$$

with

4.41 $$\begin{aligned} \hat{g}(a,k) := \int _{-\infty }^\infty g_0 \Big ( a- \frac{y^2}{2} \Big )\, e^{i k y}\, \mathrm{d}y {.} \end{aligned}$$

#### Proof

Applying Plancherel’s theorem to (4.39) gives

4.42 $$\begin{aligned} g(a,b) = \frac{1}{4b} \int _{-\infty }^\infty \frac{\mathrm{d}p}{2 \pi }\, \hat{J}(-p) \, \hat{h}_\pm (p) {,} \end{aligned}$$

where

$$\begin{aligned} \hat{J}(p)&:= \int _{-\infty }^\infty J_0(q)\, \epsilon (q)\, e^{i p q}\, \mathrm{d}q \\ \hat{h}_\pm (p)&:= \int _{-\infty }^\infty g_0'\Big ( a- \frac{q^2}{4b} \Big )\, q\, e^{i p q}\, \mathrm{d}q. \end{aligned}$$

(This relation is verified most easily by substituting the last two equations into (4.42) and using that $\int _{-\infty }^\infty e^{i p r} \mathrm{d}p = 2 \pi \delta (r)$.) The first Fourier integral was computed in Lemma 4.16. The second Fourier integral can be simplified using integration by parts,

$$\begin{aligned} \hat{h}_\pm (p) = -2b \int _{-\infty }^\infty e^{i p q}\,\frac{\mathrm{d}}{\mathrm{d}q} g_0\Big ( a- \frac{q^2}{4b} \Big )\, \mathrm{d}q = ip\,2b \int _{-\infty }^\infty g_0\Big ( a- \frac{q^2}{4b} \Big )\, e^{i p q}\, \mathrm{d}q {.} \end{aligned}$$

Introducing the new integration variable $y=q/\sqrt{2b}$ gives

$$\begin{aligned} \hat{h}_\pm (p)&= \sqrt{8}\, ip\, b^{\frac{3}{2}} \int _{-\infty }^\infty g_0 \Big ( a- \frac{y^2}{2} \Big )\, e^{i \tilde{p} y}\, \mathrm{d}y \qquad \text {with} \qquad \tilde{p} := \sqrt{2b}\, p \\&= \sqrt{8}\, ip\, b^{\frac{3}{2}} \, \hat{g}\big (a, \sqrt{2b}\, p \big ) {,} \end{aligned}$$

where in the last step we used notation (4.41).

Combining the above formulas, we obtain

$$\begin{aligned} g(a,b)&= \frac{1}{4b} \int _{\mathbb {R}\setminus [-1,1]} \frac{\mathrm{d}p}{2 \pi }\, (-2 i)\,\frac{\epsilon (p)}{\sqrt{p^2-1}}\, \sqrt{8}\, ip\, b^{\frac{3}{2}} \, \hat{g}\big (a, \sqrt{2b}\, p \big ) \\&= \frac{\sqrt{2b}}{2\pi } \int _{\mathbb {R}\setminus [-1,1]} \frac{|p|}{\sqrt{p^2-1}}\, \hat{g}\big (a, \sqrt{2b}\, p \big ) \, \mathrm{d}p \\&= \frac{\sqrt{2b}}{\pi } \int _1^\infty \frac{p}{\sqrt{p^2-1}}\, \hat{g}\big (a, \sqrt{2b}\, p \big ) \, \mathrm{d}p = \frac{1}{\pi } \int _{\sqrt{2b}}^\infty \frac{k}{\sqrt{k^2-2b}}\, \hat{g}(a, k) \, \mathrm{d}k {,} \end{aligned}$$

where in the last line we used that the integrand is even. $\square$

### Estimates of the Contour Integral

Our next goal is to estimate the contour integral in (4.41). In view of the estimate of Lemma 4.15, for the function $g_0$ it suffices to consider the explicit functions $g_0^{(1)}$ and $g_0^{(2)}$ in (4.38). In order to treat these two functions together, for a given parameter $s \in [0,1]$ we choose

4.43 $$\begin{aligned} g_0(a) = e^{3a} \,\exp \big ( s^2 \,e^{2a} \big ) {.} \end{aligned}$$

Clearly, setting $s=1$ gives the function $g_0^{(1)}$. In order to treat the function $g_0^{(2)}$, we will later integrate over the parameter $s \in [0,1]$ (see Sect. 4.14). Thus, we turn our attention to estimating the integral

$$\begin{aligned} \hat{g}(a,k) = \int _{-\infty }^\infty g_0 \Big ( a- \frac{y^2}{2} \Big )\, e^{i k y}\, \mathrm{d}y \end{aligned}$$

for the function $g_0$ as given by (4.43). In order to simplify the notation, we set

4.44 $$\begin{aligned} \lambda = s^2 \,e^{2a} {.} \end{aligned}$$

Then, the transformation

$$\begin{aligned} \exp \Big ( s^2\, e^{2 \big (a-\frac{y^2}{2} \big )} \Big ) = \exp \big ( \lambda \, e^{-y^2} \big ) \end{aligned}$$

allows us to rewrite the above integral as

4.45 $$\begin{aligned} \hat{g}(a,k) = e^{3a} \int _{-\infty }^\infty \exp \Big (-\frac{3}{2}\, y^2 + \lambda \,e^{-y^2} + i k y \Big ) \, \mathrm{d}y {.} \end{aligned}$$

We also write this integral as

4.46 $$\begin{aligned} \hat{g}(a,k)&= e^{3a} \int _{-\infty }^\infty e^{\chi (y)}\, \mathrm{d}y \qquad \text {with} \end{aligned}$$

4.47 $$\begin{aligned} \chi (y) \,&:= -\frac{3}{2}\, y^2 + \lambda \,e^{-y^2} + i k y {.} \end{aligned}$$

We want to apply a saddle-point argument. To this end, we first compute the critical points of the function $\chi$. In fact, a straightforward computation shows that there is only one critical point, which lies on the imaginary axis at

$$\begin{aligned} y = i \beta {,} \end{aligned}$$

where $\beta$ is defined implicitly by the equation

4.48 $$\begin{aligned} k = 3\, \beta + 2 \lambda \,\beta \, e^{\beta ^2} {.} \end{aligned}$$

Our strategy is to deform the integration contour such that it goes through this critical point. For simplicity, we choose the integration contour as a straight line parallel to the real axis,

$$\begin{aligned} y = \gamma (t) := t + i \beta {.} \end{aligned}$$

We thus obtain

$$\begin{aligned} \chi (y)&= \lambda \, e^{-t^2 + \beta ^2- 2 i \beta t} - 2 \lambda \,e^{\beta ^2}\,\beta ^2 -\frac{3}{2}\, \beta ^2 - \frac{3}{2}\, t^2 \\&\quad \, + 2 i\,\lambda \,e^{\beta ^2}\,\beta \, t {,} \end{aligned}$$

and thus

$$\begin{aligned} e^{\chi (y)} = A\, \exp \bigg \{ C\, e^{-t^2}\, e^{-2 i \beta t} \bigg \} \,B(t) {,} \end{aligned}$$

where we used (4.48) in order to express *k* in terms of $\beta$ and set

4.49 $$\begin{aligned} A&= \exp \Big ( - 2 \lambda \,e^{\beta ^2}\,\beta ^2 -\frac{3}{2}\, \beta ^2 \Big ) \end{aligned}$$

4.50 $$\begin{aligned} B(t)&= e^{2 i\,\lambda \,e^{\beta ^2}\,\beta \, t}\, \exp \Big (- \frac{3}{2}\, t^2 \Big ) \end{aligned}$$

4.51 $$\begin{aligned} C&= \lambda \,e^{\beta ^2} {.} \end{aligned}$$

Using this formula in (4.46), we can decompose the integral as4.524.53In order to estimate this integral, we first take the absolute value of the integrand4.54The obtained integral is estimated further in the next lemma.

#### Lemma 4.18

For any $$C \ge 0$$,

4.55 $$\begin{aligned} \int _0^\infty \exp \Big \{ C\, e^{-t^2} \Big \}\, e^{-\frac{3}{2}\, t^2}\, \mathrm{d}t \le 2\, \frac{e^C}{\sqrt{1+C}} {.} \end{aligned}$$

#### Proof

For $t \in [0,1]$, we estimate the inner exponential by a polynomial,

$$\begin{aligned} e^{-t^2} \le 1 - t^2 + \frac{t^4}{2}\, \sup _{\xi \in [0,1]} e^{-\xi ^2} \le 1 - t^2 + \frac{t^4}{2} \le 1 - \frac{t^2}{2} {.} \end{aligned}$$

This gives the estimate

4.56 $$\begin{aligned} \int _0^1 \exp \Big \{ C\, e^{-t^2} \Big \}\, e^{-\frac{3}{2}\, t^2}\, \mathrm{d}t \le \int _0^1 e^C \,\exp \Big \{ -\frac{C}{2}\,t^2 \Big \} \, e^{-\frac{3}{2}\, t^2}\, \mathrm{d}t \le \sqrt{\frac{\pi }{2}}\, \frac{e^C}{\sqrt{C+3}} {.}\nonumber \\ \end{aligned}$$

In the remaining parameter range $t \in [1,\infty )$, we use that $e^{-t^2} < e^{-1}$ to obtain

$$\begin{aligned} \int _1^\infty \exp \Big \{ C\, e^{-t^2} \Big \}\, e^{-\frac{3}{2}\, t^2}\, \mathrm{d}t \le e^{\frac{C}{e}} \int _0^\infty e^{-\frac{3}{2}\, t^2}\, \mathrm{d}t = \sqrt{\frac{\pi }{6}}\, e^{\frac{C}{e}} {.} \end{aligned}$$

For large values of *C*, contribution (4.56) clearly dominates. Since this contribution has no zeros and all contributions are bounded near $C=0$, one finds that (4.55) holds with some numerical constant on the right side. By direct inspection, one sees that this constant can be chosen equal to two. $\square$

Combining the above estimates, we obtain the following result.

#### Lemma 4.19

Integral (4.45) can be estimated by

4.57 $$\begin{aligned} \big | \hat{g}(a,k) \big |&\le \frac{c\,e^{3a}}{\sqrt{1 + \lambda \, e^{\beta ^2}}}\, e^{-h(\lambda ,k)} \qquad \text {with} \end{aligned}$$

4.58 $$\begin{aligned} h(\lambda , k) \,&:= \frac{3}{2}\, \beta ^2 - \lambda \, e^{\beta ^2} \,\Big ( 1 - 2\, \beta ^2 \Big ) {,} \end{aligned}$$

where *c* is a numerical constant, $\lambda$ is defined by (4.44), and $\beta$ is given implicitly by (4.48).

#### Proof

We combine (4.54) with (4.55) and apply the resulting inequality in (4.52). Using (4.51) gives the result. $\square$

We finally collect a few properties of the function *h* in (4.58), which will be needed in the next section.

#### Lemma 4.20

For any fixed $\lambda$,

4.59 $$\begin{aligned} h(\lambda , k)&= -\frac{3}{2} \beta ^2 - k \,\Big ( \frac{1}{2\beta } - \beta \Big ) + \frac{3}{2} \end{aligned}$$

4.60 $$\begin{aligned} \frac{\partial h(\lambda , k)}{\partial k}&= \beta \end{aligned}$$

4.61 $$\begin{aligned} \frac{\partial h(\lambda , k)}{\partial \lambda }&= -e^{\beta ^2} \end{aligned}$$

4.62 $$\begin{aligned} \frac{\partial ^2 h(\lambda , k)}{\partial \lambda ^2}&>0 {,} \end{aligned}$$

where *k* is given via (4.48) in terms of $\lambda$ and $\beta$. Moreover, for any $k>\tilde{k}$,

4.63 $$\begin{aligned} h(\lambda , k) \ge h(\lambda , \tilde{k}) + \tilde{\beta }\, \big (k-\tilde{k} \big ){.} \end{aligned}$$

#### Proof

Relation (4.59) follows immediately from (4.58) and (4.48). Next, a direct computation using again (4.58) and (4.48) yields

4.64 $$\begin{aligned} \frac{\partial h}{\partial \beta }&= 3\, \beta + 2 \lambda \, \beta \, e^{\beta ^2} + 4 \lambda \, \beta ^3\, e^{\beta ^2} \end{aligned}$$

4.65 $$\begin{aligned} \frac{\partial k}{\partial \beta }&= 3 + 2 \lambda \, e^{\beta ^2} + 4 \lambda \, \beta ^2\, e^{\beta ^2} {.} \end{aligned}$$

Combining these equations with the chain rule gives (4.60).

In order to compute the partial derivatives with respect to $\lambda$, we first compute the total derivative of (4.48) for fixed *k*,

$$\begin{aligned} 0 = \mathrm{d}k = 2 \beta \, e^{\beta ^2} \, \mathrm{d}\lambda + \Big ( 3 + 2 \lambda e^{\beta ^2} (1+2 \beta ^2) \Big )\, \mathrm{d}\beta {.} \end{aligned}$$

Hence,

4.66 $$\begin{aligned} \frac{\mathrm{d} \beta }{\mathrm{d}\lambda } = -\frac{2 \beta \, e^{\beta ^2}}{3 + 2 \lambda e^{\beta ^2} (1+2 \beta ^2)} {.} \end{aligned}$$

This formula shows in particular that, for fixed *k*, the function $\beta$ is monotone decreasing in $\lambda$. On the other hand, a direct computation using (4.59) and again (4.48) gives

4.67 $$\begin{aligned} \frac{\partial h}{\partial \beta } = \frac{3 + 2 \lambda e^{\beta ^2} (1+2 \beta ^2)}{2\, \beta }. \end{aligned}$$

(The partial derivative is again computed for fixed *k*.) Taking the product of (4.66) and (4.67) gives (4.61). Differentiating once again and using that $\beta$ is monotone decreasing gives (4.62).

In order to derive (4.63), we first note that from (4.48) or (4.65) it follows that, for fixed $\lambda$, the function $\beta$ is monotone increasing in *k*. Therefore,

$$\begin{aligned} h(\lambda , k) - h(\lambda , \tilde{k}) = \int _{\tilde{k}} ^k \frac{\partial h(\lambda , \hat{k})}{\partial \hat{k}}\, \mathrm{d}\hat{k} \overset{(4.60)}{=} \int _{\tilde{k}}^k \hat{\beta }\, \mathrm{d}\hat{k} \ge \tilde{\beta }\, \big (k-\tilde{k} \big ){.} \end{aligned}$$

This concludes the proof. $\square$

### Estimate of $g^{(1)}$

The goal of this section is to estimate the solution of the Goursat problem *g*(*a*, *b*) in (4.35) with initial data $g_0^{(1)}$ as in (4.38). Our starting point is the estimate of Lemma 4.19, where we set $s=1$ (cf. (4.43) and (4.38)). Our task is to estimate integral (4.40). To this end, we need to distinguish different cases:

**Case (A)**: $0 \le \beta < 1$. In view of (4.48), this corresponds to the range for *k*

4.68 $$\begin{aligned} k < k_0 := 3 + 2 e\, \lambda {.} \end{aligned}$$

In this case, we can estimate $\beta$ in terms of *k* by

4.69 $$\begin{aligned} k \le (3 + 2 e \lambda )\, \beta {,}\qquad \beta \ge \frac{k}{3 + 2 e \lambda } {.} \end{aligned}$$

**Case (B)**: $\beta \ge 1$. In view of (4.48), this corresponds to the range for *k*

$$\begin{aligned} k \ge k_0 = 3 + 2 e\, \lambda {.} \end{aligned}$$

In order to express $\beta$ in terms of *k*, we distinguish two sub-cases. We set

4.70 $$\begin{aligned} {{\,\mathrm{Im}\,}}y_1 := \left\{ \begin{array}{ll} \displaystyle \sqrt{-\log \frac{2 \lambda }{3}} &{} \text {if } \displaystyle \lambda <\frac{3}{2 e} \\ 1 &{} \text {if } \displaystyle \lambda \ge \frac{3}{2 e} {.} \end{array} \right. \end{aligned}$$

**Case (B1)**: $1 \le \beta < {{\,\mathrm{Im}\,}}y_1$. Clearly, this case only occurs if ${{\,\mathrm{Im}\,}}y_1 >1$, which by (4.70) implies that

$$\begin{aligned} \lambda < \frac{3}{2e}{.} \end{aligned}$$

Moreover,

$$\begin{aligned} \lambda \, e^{\beta ^2} \le \lambda \, e^{{{\,\mathrm{Im}\,}}^2 y_1} = \frac{3}{2} {.} \end{aligned}$$

Using (4.48), we obtain

$$\begin{aligned} k < k_1 \,\,&:= 3\, {{\,\mathrm{Im}\,}}y_1 + 2 \lambda \,{{\,\mathrm{Im}\,}}y_1\, e^{{{\,\mathrm{Im}\,}}^2 y_1} \\&= \sqrt{-\log (2\lambda /3)} \,\big ( 3 + 2 \lambda \, e^{-\log (2\lambda /3)} \big ) = 6\, \sqrt{-\log (2 \lambda /3)} = 6\, {{\,\mathrm{Im}\,}}y_1{.} \end{aligned}$$

Therefore, we can estimate (4.48) from above and below by

4.71 $$\begin{aligned} k - 3 \beta \le 3\, \beta \end{aligned}$$

4.72 $$\begin{aligned} 3 \beta \le k \le 6\, \beta {,}\qquad \frac{k}{6} \le \beta \le \frac{k}{3}{.} \end{aligned}$$

**Case (B2)**: $\beta \ge \max \{1,{{\,\mathrm{Im}\,}}y_1\}$. In this case,

$$\begin{aligned} \lambda \, e^{\beta ^2} \ge \lambda \, e^{{{\,\mathrm{Im}\,}}^2 y_1} = \frac{3}{2} {,} \end{aligned}$$

making it possible to estimate (4.48) by

4.73 $$\begin{aligned} k - 3 \beta \ge 3\, \beta \end{aligned}$$

4.74 $$\begin{aligned} k = 3\, \beta + 2 \lambda \,\beta \, e^{\beta ^2} \le 4 \lambda \,\beta \, e^{\beta ^2} {.} \end{aligned}$$

The resulting inequality can be estimated with the help of Lambert’s *W*-function. Indeed, taking the square of the above inequality,

$$\begin{aligned} \frac{k^2}{8 \lambda ^2} \le 2 \beta ^2\, e^{2 \beta ^2} {,} \end{aligned}$$

one obtains (for details see [22,  Eq. 4.13.1])

$$\begin{aligned} \beta ^2 \ge \frac{1}{2}\, W\Big (\frac{k^2}{8 \lambda ^2} \Big ) {.} \end{aligned}$$

In the region $k \ge k_0$ under consideration, the argument of the *W*-function is larger than $e^2/2 \approx 3.69$, making it possible to use the inequalities

$$\begin{aligned} \log x - \log \big ( \log x \big ) \le W(x) \le \log x \qquad \text {if } x\ge \frac{e^2}{2}{.} \end{aligned}$$

We thus obtain the estimate

4.75 $$\begin{aligned} 2\,\beta ^2 \ge \log \Big (\frac{k^2}{8 \lambda ^2} \Big ) - \log \bigg ( \log \Big (\frac{k^2}{8 \lambda ^2} \Big ) \bigg ) {.} \end{aligned}$$

**Fig. 2:**
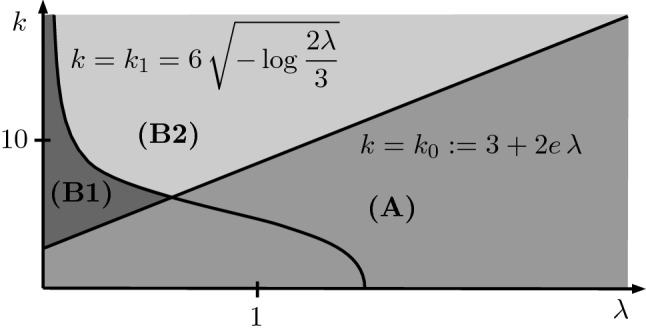
Different cases in the $k\lambda$-plane

The different cases are shown schematically in Fig. 2.

We now state the main result of this section. For notational convenience,

$$\begin{aligned} A \lesssim B \qquad \text {stands for} \qquad A \le c\, B \end{aligned}$$

for a suitable numerical constant $c>0$ (which does not depend on any parameters).

#### Proposition 4.21

The function *g*(*a*, *b*) in (4.40) is bounded by

4.76 $$\begin{aligned} |g(a,b)|&\lesssim e^{3a} \, e^{-h(\sqrt{2b},\lambda )} \end{aligned}$$

4.77 $$\begin{aligned}&= e^{3a} \, \exp \bigg ( \frac{3}{2} \beta ^2 + \sqrt{2b} \,\Big ( \frac{1}{2\beta } - \beta \Big ) \bigg ) {,} \end{aligned}$$

where *h* is the function (4.58) and $\beta$ is determined implicitly by (4.48) for $k=\sqrt{2b}$, i.e.,

4.78 $$\begin{aligned} \sqrt{2b} = 3\, \beta + 2 \lambda \,\beta \, e^{\beta ^2} \end{aligned}$$

(and $\lambda$ is given in terms of *a* by (4.44)). More explicitly, $\beta$ is bounded from below by

4.79 $$\begin{aligned} \beta \ge \left\{ \begin{array}{cl} \displaystyle \frac{\sqrt{2b}}{3 + 2 e \lambda } &{} \text {in case } (\textbf{A}) \\ \displaystyle \frac{\sqrt{2b}}{6} &{} \text {in case } (\textbf{B1}) \\ \displaystyle \frac{1}{\sqrt{2}} \,\sqrt{\log \Big (\frac{b}{4 \lambda ^2} \Big ) - \log \bigg ( \log \Big (\frac{b}{4 \lambda ^2} \Big )} &{} \text {in case } (\textbf{B2}) {,} \end{array} \right. \end{aligned}$$

with the cases as above with $k=\sqrt{2b}$ and $\beta$ given by (4.78).

We now enter the detailed estimates. The proof of this proposition will be completed at the end of this section. Our strategy is to estimate the *k*-integral in the different regions separately. To this end, we decompose the range of integration as

$$\begin{aligned} (\sqrt{2b}, \infty ) = I_{(A)} \dot{\cup } I_{(B1)} \dot{\cup } I_{(B2)} \end{aligned}$$

with

$$\begin{aligned} I_{A} = \big (\sqrt{2b}, k_0 \big ){,}\quad I_{B1} = \big [\max \{\sqrt{2b}, k_0\}, k_1\big ) {,}\quad I_{B2} = \big [\max \{\sqrt{2b}, k_0, k_1\}, \infty \big ) {.} \end{aligned}$$

We begin with an estimate in case **(A)**.

#### Lemma 4.22

The following inequality holds,

$$\begin{aligned} g_{A} := \int _{I_{A}} \frac{k}{\sqrt{k^2-2b}}\, \big |\hat{g}(a, k)\big | \, \mathrm{d}k \le e^{3a} \,\exp \bigg ( \sqrt{2b} \,\Big ( \frac{1}{2\beta } - \beta \Big ) \bigg ) {,} \end{aligned}$$

where $\beta$ is chosen according to (4.78).

#### Proof

Using the inequality $0 \le \beta <1$, we estimate (4.57) by

$$\begin{aligned} \big | \hat{g}(a,k) \big | \lesssim \frac{e^{3a}}{\sqrt{1 + \lambda }}\, \exp \bigg ( \lambda \, e^{\beta ^2} \,\Big ( 1 - 2\, \beta ^2 \Big ) \bigg ) {.} \end{aligned}$$

Setting $x = \beta ^2$, the last exponent involves the function

4.80 $$\begin{aligned} f(x):= e^x\, (1-2x) {,} \end{aligned}$$

whose first and second derivatives are negative,

$$\begin{aligned} f'(x) = -e^x \,\big ( 1+2x \big )< 0 \qquad \text {and} \qquad f''(x) = -e^x \,\big ( 3+2x \big ) < 0 {.} \end{aligned}$$

In particular, the function *f* is concave. Therefore, choosing $\tilde{x}$, for all $x>\tilde{x}$,

$$\begin{aligned} f(x) \le f\big ( \tilde{x} \big ) + f'(\tilde{x}) \, (x-\tilde{x}) {.} \end{aligned}$$

As a consequence,

$$\begin{aligned} \big | \hat{g}(a,k) \big | \lesssim \frac{e^{3a}}{\sqrt{1 + \lambda }}\, \exp \bigg ( \lambda \, f\big ( \tilde{\beta }^2 \big ) + \lambda \, f'\big ( \tilde{\beta }^2 \big ) \, \big ( \beta ^2 - \tilde{\beta }^2 \big ) \bigg ) {,} \end{aligned}$$

where we choose $\tilde{\beta }$ such that (4.78) holds. Applying (4.65) and (4.69), we obtain the estimate

$$\begin{aligned} \beta ^2 - \tilde{\beta }^2&= \int _{\tilde{k}}^k \frac{\mathrm{d}}{\mathrm{d}k'} \beta ^2\, \mathrm{d}k' = \int _{\tilde{k}}^k \frac{2 \beta }{3 + 2 \lambda \, e^{\beta ^2} + 4 \lambda \, \beta ^2\, e^{\beta ^2}} \, \mathrm{d}k' \\&\ge \frac{2}{(3 + 2 e \lambda )(3 + 6 e \lambda )} \int _{\tilde{k}}^k k'\, \mathrm{d}k' \ge \frac{1}{(3 + 6 e \lambda )^2} \, \big ( k^2 - 2b \big ) {,} \end{aligned}$$

where in the last line we also used that $\beta <1$. We thus obtain the estimate

$$\begin{aligned} \big | \hat{g}(a,k) \big | \lesssim \frac{e^{3a+\lambda f(\tilde{\beta }^2)}}{\sqrt{1 + \lambda }}\; \exp \bigg ( -\frac{\lambda \,|f'(\tilde{\beta }^2)|}{(3 + 2 e \lambda )^2} \, \big (k^2 - 2b \big ) \bigg ) {.} \end{aligned}$$

Now, we can estimate the integral by

$$\begin{aligned} g_A&\le \int _{\sqrt{2b}}^{k_0} \frac{k}{\sqrt{k^2-2b}}\, \big | \hat{g}(a,k) \big | \,\mathrm{d}k = \left\{ \begin{array}{c} z = \sqrt{k^2 - 2b} \\ z \,\mathrm{d}z = k \, \mathrm{d}k \end{array} \right\} \\&= \int _0^{\sqrt{k_0^2-2b}} \big | \hat{g}(a,\sqrt{z^2+2b}) \big | \,\mathrm{d}z \\&\lesssim \frac{e^{3a+\lambda f(\tilde{\beta }^2)}}{\sqrt{1 + \lambda }} \int _0^\infty \exp \bigg ( -\frac{\lambda \,|f'(\tilde{\beta }^2)|}{(3 + 6 e \lambda )^2} \, z^2 \bigg )\,\mathrm{d}z \\&\lesssim \frac{e^{3a+\lambda f(\tilde{\beta }^2)}}{\sqrt{1 + \lambda }}\, \frac{3+6 e \lambda }{\sqrt{\lambda \,|f'(\tilde{\beta }^2)|}} \lesssim \frac{e^{3a+\lambda f(\tilde{\beta }^2_0)}}{|f'(\tilde{\beta }^2)|} \lesssim e^{3a+\lambda f(\tilde{\beta }^2)}{,} \end{aligned}$$

where in the last line we computed the Gaussian integral and used that $\lambda$ and $|f'|$ are bounded from below. Applying (4.80) and using that $\tilde{\beta }<1$ give the result (where for notational convenience, in the statement of the lemma we omitted the tilde). $\square$

In order to estimate the integral in case **(B)**, we consider a general integral

4.81 $$\begin{aligned} g_B := \int _{\hat{k}}^{k_2} \frac{k}{\sqrt{k^2-2b}}\, \big |\hat{g}(a, k)\big | \, \mathrm{d}k \end{aligned}$$

with $\hat{k} = \max \{ k_0, \sqrt{2b} \}$ and $k_2 \ge \hat{k}$. In this case, we write the estimate of Lemma 4.19 using (4.63) as

4.82 $$\begin{aligned} \big | \hat{g}(a,k) \big |&\lesssim \frac{e^{3a}}{\sqrt{1 + \lambda \, e^{\beta ^2}}}\, e^{-h(\lambda ,\hat{k})} \, \exp \Big ( -\hat{\beta }\, \big (k-\hat{k} \big ) \Big ) \nonumber \\&\lesssim \frac{e^{3a}}{\sqrt{1 + \lambda \, e^{\tilde{\beta }^2}}}\, e^{-h(\lambda ,\hat{k})} \, \exp \Big ( -\hat{\beta }\, \big (k-\hat{k} \big ) \Big ) {,} \end{aligned}$$

where in the last step we again used that $\beta$ is monotone increasing in *k*. In this inequality, the *k*-dependence is given simply by a decaying exponential. Therefore, we may replace the upper limit of integration $k_2$ in (4.81) by $\infty$. Thus, it remains to estimate the integral

$$\begin{aligned} \int _{\hat{k}}^\infty \frac{k}{\sqrt{k^2 - 2b}}\, e^{-\beta \, (k-\hat{k})} \, \mathrm{d}k {.} \end{aligned}$$

In preparation, we shift the integration variable such as to obtain an integral over the interval $[\sqrt{2b}, \infty )$,

4.83 $$\begin{aligned}&\int _{\hat{k}}^\infty \frac{k}{\sqrt{k^2 - 2b}}\, e^{-\beta \, (k-\hat{k})} \, \mathrm{d}k = \left\{ k' = k - \ell \text { with } \ell :=\hat{k} - \sqrt{2b} \ge 0 \right\} \nonumber \\&\quad =\int _{\sqrt{2b}}^\infty \frac{k' + \ell }{\sqrt{(k'+\ell )^2 - 2b}}\, e^{-\beta \, \big ( k'-\sqrt{2b} \big )} \, \mathrm{d}k' \nonumber \\&\quad \le \int _{\sqrt{2b}}^\infty \frac{k'}{\sqrt{k'^2 - 2b}}\, e^{-\beta \, \big ( k'-\sqrt{2b} \big )} \, \mathrm{d}k' {,} \end{aligned}$$

where in the last step we used that the integrand is monotone decreasing in $\ell$.

#### Lemma 4.23

For any parameters $b \ge 0$ and $d > 0$,

$$\begin{aligned} \int _{\sqrt{2b}}^\infty \frac{k}{\sqrt{k^2-2b}}\, e^{-d\, \big (k-\sqrt{2b} \big )}\, \mathrm{d}k&\lesssim \frac{b^\frac{1}{4}}{\sqrt{d}} + \frac{1}{d} {.} \end{aligned}$$

#### Proof

Introducing the variable *z* by

$$\begin{aligned} z(k) := \sqrt{ \frac{k^2}{2b} - 1} {,}\qquad k = \sqrt{2b}\, \sqrt{z^2+1} {,}\qquad k\, \mathrm{d}k = 2b\, z\, \mathrm{d}z {,} \end{aligned}$$

we obtain

$$\begin{aligned} \int _{\sqrt{2b}}^\infty \frac{k}{\sqrt{k^2-2b}}\, e^{-\mathrm{d}k}\, \mathrm{d}k&= \int _0^\infty \frac{1}{\sqrt{2b}\, z}\, e^{-C \,\sqrt{z^2+1}}\,2b\, z\, \mathrm{d}z\\&= \sqrt{2b} \int _0^\infty e^{-C \,\sqrt{z^2+1}}\, \mathrm{d}z \end{aligned}$$

with

$$\begin{aligned} C := d\, \sqrt{2b} \ge \sqrt{2} {.} \end{aligned}$$

In order to estimate the integral further, we consider two cases:

- $$0 \le z \le 1$$: The inequalities
  $$\begin{aligned} 1+\frac{z^2}{3} \le \sqrt{z^2+1} \le \sqrt{2} \end{aligned}$$
  give rise to the estimate
  $$\begin{aligned} \int _{0}^1 e^{-C \,\sqrt{z^2+1}}\, \mathrm{d}z&\le e^{-C} \int _{0}^1 e^{-\frac{C}{3}\, z^2}\, \mathrm{d}z \\&\le e^{-C} \int _{0}^\infty e^{-\frac{C}{3}\, z^2}\, \mathrm{d}z = \frac{\sqrt{3 \pi }}{2}\, \frac{e^{-C}}{\sqrt{C}} {.} \end{aligned}$$
- $1 \le z$: In this case,
  $$\begin{aligned} \sqrt{2} + \frac{1}{\sqrt{2}}\, (z-1) \le \sqrt{z^2+1} \le \sqrt{2}\,z {,} \end{aligned}$$
  and thus
  $$\begin{aligned} \int _1^\infty e^{-C \,\sqrt{z^2+1}}\, \mathrm{d}z&\le e^{-C\, \sqrt{2}}\int _1^\infty e^{-\frac{C}{\sqrt{2}}\, (z-1)}\, \mathrm{d}z = e^{-C\, \sqrt{2}} \; \frac{\sqrt{2}}{C} {.} \end{aligned}$$

Collecting all the contributions gives the result. $\square$

#### Proof of Proposition 4.21

Applying Lemma 4.23 in (4.81), (4.82) and using (4.83), we obtain

$$\begin{aligned} |g_B| \lesssim \frac{e^{3a}}{\sqrt{1 + \lambda \, e^{\hat{\beta }^2}}}\, e^{-h(\lambda ,\hat{k})} \, \bigg ( \frac{b^{\frac{1}{4}}}{\sqrt{\hat{\beta }}} + \frac{1}{\hat{\beta }} \bigg ) {.} \end{aligned}$$

The terms in the denominator can be simplified because, using (4.48),

$$\begin{aligned} \big ( 1 + \lambda \, e^{\hat{\beta }^2} \big )\, \hat{\beta } \simeq \big ( 3 + 2\lambda \, e^{\hat{\beta }^2} \big )\, \hat{\beta } = \hat{k} {.} \end{aligned}$$

Applying (4.59), we obtain the estimate

4.84 $$\begin{aligned} |g_B| \le e^{3a}\, \exp \bigg ( \frac{3}{2}\, \hat{\beta }^2 + \frac{\hat{k}}{2\, \hat{\beta }} - \hat{\beta }\, \hat{k} \bigg ) \, \bigg ( \frac{b^{\frac{1}{4}}}{\sqrt{\hat{\beta }}} + 1\bigg ) {,} \end{aligned}$$

where we simplified the last summand inside the last brackets by using the inequality $\hat{\beta } \ge 1$. This concludes the estimates in case **(B)**.

Next, we need to add the integrals in the different regions. Noting that $\beta <1$ in case **(A)**, the estimate of Lemma 4.22 agrees with the estimate in (4.84) if we choose $\hat{k}=\sqrt{2b}$. Noting that, in view of (4.60), the argument of the exponent is decreasing in $\hat{k}$, it suffices to consider the contribution in the region corresponding to the case determined by $k=\sqrt{2b}$. This gives (4.76). The lower bounds in (4.79) were derived in (4.69), (4.72), and (4.75). $\square$

### Estimate of $g^{(2)}$

We now come to the estimate of the solution of the Goursat problem *g*(*a*, *b*) in (4.35) with initial data $g_0^{(2)}$ as in (4.38). Our task is to estimate the *s*-integral in (4.38). In view of (4.44), this corresponds to integrating $\lambda$ along a straight line

$$\begin{aligned} \lambda = s^2\, \lambda _0 \qquad \text {with} \qquad s \in [0,1] \text { and } \lambda _0 := e^{2a} {.} \end{aligned}$$

More precisely, our task is to estimate the integral

$$\begin{aligned} \int _0^1 |g(a,b)|\big |_{\lambda = s^2 \lambda _0}\, \mathrm{d}s \end{aligned}$$

with |*g*(*a*, *b*)| as estimated in (4.76) and $\beta$ as given implicitly by (4.78).

According to (4.62), the function $h(., \sqrt{2b})$ is convex. Hence,

$$\begin{aligned} h\big ( \lambda , \sqrt{2b}) \ge h\big (\lambda _0, \sqrt{2b} \big ) + \frac{\partial h\big (\lambda , \sqrt{2b}\big )}{\partial \lambda }\bigg |_{\lambda =\lambda _0} \, (\lambda -\lambda _0) {.} \end{aligned}$$

As a consequence,

$$\begin{aligned} \int _0^1&|g(a,b)|\Big |_{\lambda = s^2 \lambda _0}\, \mathrm{d}s \lesssim e^{3a} \int _0^1 e^{-h\big ( s^2 \lambda _0, \sqrt{2b}\big )}\, \mathrm{d}s \\&\le e^{3a} \int _0^1 e^{-h\big (\lambda _0, \sqrt{2b} \big ) - \partial _\lambda h\big (\lambda _0, \sqrt{2b} \big )\, \lambda _0\, (s^2-1)}\, \mathrm{d}s \\&= e^{3a}\, e^{-h\big (\lambda _0, \sqrt{2b} \big )} \int _0^1 e^{-\partial _\lambda h\big (\lambda _0, \sqrt{2b} \big )\, \lambda _0\, (s^2-1)}\, \mathrm{d}s \\&=e^{3a}\, e^{-h\big (\lambda _0, \sqrt{2b} \big )}\, \frac{\sqrt{\pi }}{2} \frac{e^{-\nu }}{\sqrt{\nu }}\, \text {Erfi}(\nu ) \end{aligned}$$

with

$$\begin{aligned} \nu := -\partial _\lambda h\big (\sqrt{2b}, \lambda _0 \big )\, \lambda _0 \overset{(4.61)}{=} \lambda \,e^{\beta ^2}\big |_{\lambda =\lambda _0} {,} \end{aligned}$$

where $\text {Erfi}$ is the imaginary error function.

Using this result in the formula of Lemma 4.15, we obtain the following result:

#### Proposition 4.24

The solution of the Goursat problem (4.35) with initial data (4.33) is bounded by

$$\begin{aligned} |g(a,b)|&\lesssim e^{3a} \, \exp \bigg ( \frac{3}{2} \beta ^2 + \sqrt{2b} \,\Big ( \frac{1}{2\beta } - \beta \Big ) \bigg ) \, \sqrt{\frac{e^{-\nu }}{\sqrt{\nu }}\, \text {Erfi}(\nu ) } {,} \end{aligned}$$

where $\beta$ and $\nu$ are given by

$$\begin{aligned} \sqrt{2b}&= 3\, \beta + 2 e^{2a}\,\beta \, e^{\beta ^2} \\ \nu&= e^{2a} \,e^{\beta ^2} {.} \end{aligned}$$

We finally state our results in a way compatible with Theorem 1.1.

#### Corollary 4.25

There is a numerical constant $c>0$ such that the function $R(\varepsilon , \omega )$ in (1.2) can be chosen as

4.85 $$\begin{aligned} R(\varepsilon , \omega ) = c\,\exp \bigg ( \frac{3}{2} \beta ^2 + 2 \,\sqrt{|\log \varepsilon |}\, \Big ( \frac{1}{2\beta } - \beta \Big ) \bigg ) \, \sqrt{\frac{e^{-\nu }}{\sqrt{\nu }}\, \text {Erfi}(\nu )} \end{aligned}$$

with $\beta$ and $\nu$ as given implicitly by

4.86 $$\begin{aligned} 2 \,\sqrt{|\log \varepsilon |}&= 3\, \beta + 8 \omega \,\beta \, e^{\beta ^2} \end{aligned}$$

4.87 $$\begin{aligned} \nu&= 4 \omega \,e^{\beta ^2} {.} \end{aligned}$$

#### Proof

We use the result of Proposition 4.24 in Proposition 4.8 and apply (4.28).

$$\square $$

We conclude this section with a brief discussion of our final result. Clearly, due to the implicit definition of $\beta$ and $\nu$ via (4.86) and (4.87), the estimate of Corollary 4.25 is rather involved. Its meaning can be revealed by considering various limiting cases. For brevity, we here only consider a particular case which explains why our last estimate goes beyond the previous estimates in Theorems 4.10 and 4.13. To this end, we consider the limiting case

4.88 $$\begin{aligned} |\log \varepsilon | \simeq \sqrt{\omega } \qquad \text {and} \qquad \omega \rightarrow \infty {.} \end{aligned}$$

In this limiting case, the first exponential inside the curly brackets in (4.30) is bounded from below, implying that the right side of (4.30) tends to infinity as $\omega \rightarrow \infty$. Thus, Theorem 4.13 does not give any information on the limiting case (4.88). On the other hand, relation (4.86) implies that $\beta \sim \omega ^{-3/4} \rightarrow 0$. Consequently, (4.87) implies that $\nu \sim \omega$, giving rise to an exponential decay in (4.85). We conclude that Corollary 4.25 allows us to estimate $R(\varepsilon , \omega )$ in the limiting case (4.88), although Theorem 4.13 fails.

## The $3+1$-Dimensional Case

Let $B_1 \subset \mathbb {R}^3$ be the unit ball. We consider the Cauchy problem for the scalar wave equation with smooth, compactly supported initial data in $B_1$,

$$\begin{aligned} \left\{ \begin{array}{l} (\partial _t^2 - \Delta _{\mathbb {R}^3}) \phi (t,\textbf {x}) = 0 \\ \phi |_{t=0} = \phi _0 \in C^\infty _0(B_1) {,} \qquad \partial _t \phi |_{t=0} = \phi _1 \in C^\infty _0(B_1) {.} \end{array} \right. \end{aligned}$$

We denote the energy of the solution by

5.1 $$\begin{aligned} E(\phi ) := \frac{1}{2} \int _{B_1} \Big ( \big |\partial _t \phi (0,\textbf {x}) \big |^2 + \big | \nabla \phi (0,\textbf {x})\big |^2 \Big )\, \mathrm{d}^3x {.} \end{aligned}$$

In order to write the solution in an explicit form, it is useful to form the spatial Fourier transform defined by

$$\begin{aligned} \hat{\phi }(t,\textbf {k}) = \int _{B_1} \phi (t,\textbf {x}) \, e^{-i \textbf {k} \textbf {x}}\, \mathrm{d}^3x {.} \end{aligned}$$

Indeed, as is verified by direct computation, we have

$$\begin{aligned} \hat{\phi }(t,\textbf {k}) = \hat{\phi }_+(t,\textbf {k}) + \hat{\phi }_-(t,\textbf {k}) \end{aligned}$$

with

5.2 $$\begin{aligned} \hat{\phi }_\pm (t,\textbf {k}) := \frac{1}{2} \, e^{-i \omega t}\Big ( \hat{\phi }_0(\textbf {k}) \pm \frac{i}{\omega }\, \hat{\phi }_1(\textbf {k}) \Big ) {,} \end{aligned}$$

where we set

$$\begin{aligned} \omega = \omega (\textbf {k}) := |\textbf {k}| {.} \end{aligned}$$

The solutions $\phi _\pm$ are the components of positive and negative frequency, respectively. We again express the energy with the help of Plancherel’s theorem as an integral in momentum space:

### Lemma 5.1

Energy (5.1) can be written as

5.3 $$\begin{aligned} E(\phi ) = E(\phi _+) + E(\phi _-) \qquad \text {with} \qquad E_\pm (\phi ) := \int _{\mathbb {R}^3} \frac{\mathrm{d}^3k}{(2 \pi )^3} \,\omega ^2 \, \big | \hat{\phi }_\pm (t, \textbf {k}) \big |^2{.}\nonumber \\ \end{aligned}$$

### Proof

A direct computation using Plancherel’s theorem gives

$$\begin{aligned} E(\phi )&= \frac{1}{2} \int _{\mathbb {R}^3} \frac{\mathrm{d}^3k}{(2 \pi )^3} \,\Big ( \omega ^2\, \big |\hat{\phi }_0(\textbf {k})\big |^2 + \big |\hat{\phi }_1(\textbf {k}) \big |^2 \Big ) \\&= \int _{\mathbb {R}^3} \frac{\mathrm{d}^3k}{(2 \pi )^3} \;\omega ^2\,\Big ( \big |\hat{\phi }_+(t, \textbf {k})\big |^2 + \big |\hat{\phi }_-(t, \textbf {k})\big |^2 \Big ) {,} \end{aligned}$$

concluding the proof. $\square$

Due to spherical symmetry of the problem, we can expand the functions in spherical harmonics, in both position and momentum space. For the initial data, we obtain in polar coordinates $(r, \vartheta , \varphi )$ the representations

$$\begin{aligned} \phi _a(\textbf {x}) = \sum _{l=0}^\infty \sum _{m=-l}^l Y_{lm}(\vartheta , \varphi )\, \phi _a^{lm}(r) \qquad \text {with} \qquad a \in \{0,1\} {.} \end{aligned}$$

Similarly, in momentum space we obtain the representations

5.4 $$\begin{aligned} \hat{\phi }_a(\textbf {k}) = \sum _{l=0}^\infty \sum _{m=-l}^l Y_{lm}(\vartheta , \varphi )\, \hat{\phi }_a^{lm}(\omega ) {,} \end{aligned}$$

now in polar coordinates $(\omega =|\textbf {k}|, \vartheta , \varphi )$ in momentum space. Since Fourier transformation preserves angular momentum, it follows that the Fourier transformation of $Y_{lm} \phi _a^{lm}$ is $Y_{lm} \hat{\phi }_a^{lm}$. Moreover, being the Fourier transform of functions supported in $B_1(0)$, the functions $\hat{\phi }_a$ are real analytic. Therefore, they can be expanded in a Taylor series about $\textbf {k}=0$. We write the resulting expansion as

$$\begin{aligned} \hat{\phi }_a(\textbf {k}) = \sum _{l=0}^\infty \sum _{m=-l}^l Y_{lm}(\vartheta , \varphi ) \sum _{p=0}^\infty c^{lm}_{a,p}\, \omega ^{l+2p}{.} \end{aligned}$$

In order to explain this formula, we note that the product $Y_{lm}(\vartheta , \varphi )\,\, \omega ^l$ is a homogeneous polynomial in $\textbf {k}$ of degree *l*. Therefore, in order to have a smooth function also in $\omega$, the remaining series expansion must involve only even powers of $\omega$. Using these expansions in (5.2), we obtain

5.5 $$\begin{aligned}&\omega \, \hat{\phi }_\pm (t, \textbf {k}) = e^{\mp i \omega t} \sum _{l=0}^\infty \sum _{m=-l}^l Y_{lm}(\vartheta , \varphi )\, \hat{h}^{lm}_\pm (\omega ) \quad \text {with} \end{aligned}$$

5.6 $$\begin{aligned}&\hat{h}^{lm}_\pm (\omega ) := \sum _{n=l}^\infty a_n^{lm}\, \omega ^n {,} \end{aligned}$$

where the coefficients are given by

5.7 $$\begin{aligned} a^{lm}_{l+2p} = \pm \frac{i}{2}\, c^{lm}_{1,p} \qquad \text {and} \qquad a^{lm}_{l+2p+1} = \frac{1}{2}\, c^{lm}_{0,p} {.} \end{aligned}$$

We point out that, in contrast to the $1+1$-dimensional case, here a parity splitting is not necessary because it is already contained in the expansion in spherical harmonics. (Indeed, even *l* corresponds to even parity and odd *l* corresponds to odd parity.)

In analogy to  (4.11), the energies can be expressed in terms of the functions $\hat{h}^{lm}_\pm$ in (5.6):

### Lemma 5.2

The energies of the positive- and negative-frequency components of $\phi$ in (5.1) can be written as

$$\begin{aligned} E(\phi _\pm ) = \sum _{l=0}^\infty \sum _{m=-l}^l E^{lm}(\phi _\pm ) \end{aligned}$$

with

5.8 $$\begin{aligned} E^{lm}(\phi _\pm )=E(Y_{lm}\, \phi _\pm ^{lm})= \frac{1}{2 \pi ^2}\int _0^\infty \bigg | \sum _{n=l}^\infty a^{lm}_n\, \omega ^n \bigg |^2\, \omega ^2\, \mathrm{d}\omega \, \end{aligned}$$

### Proof

Using expansion (5.5) in (5.3) and using the orthonormality of the spherical harmonics, we obtain

$$\begin{aligned} E_\pm (\phi )&= \int _{\mathbb {R}^3} \frac{\mathrm{d}^3k}{(2 \pi )^3} \,\omega ^2 \, \big | \hat{\phi }_\pm (t, \textbf {k}) \big |^2 \\&= \sum _{l=0}^\infty \sum _{m=-l}^l \frac{4 \pi }{(2 \pi )^3} \int _0^\infty \bigg | \sum _{n=l}^\infty a_n^{lm} \omega ^n \bigg |^2 \,\omega ^2\,\mathrm{d}\omega {.} \end{aligned}$$

This concludes the proof. $\square$

We point out that there are two major differences compared to the $1+1$-dimensional situation: First, the sum over *n* in (5.6) starts at $n=l$. This is because the contributions of higher angular momentum vanish to higher order at $k=0$. Second and more importantly, the additional factor $\omega ^2$ in (5.8) is a result of the three-dimensional integration in polar coordinates in momentum space.

The next lemma gives an estimate of each Taylor coefficient in momentum space. It can be regarded as the $3+1$-dimensional analog of Lemma 2.1.

### Lemma 5.3

Let $\phi \in C^\infty _0(B_1)$ with angular decomposition

$$\begin{aligned} \phi (x) = \sum _{l=0}^\infty \sum _{m=-l}^l Y_{lm}(\vartheta , \varphi )\, \phi ^{lm}(r) {.} \end{aligned}$$

Then, its Fourier transform has a Taylor-series representation

$$\begin{aligned} \hat{\phi }(k) = \sum _{l=0}^\infty \sum _{m=-l}^l Y_{lm}(\vartheta , \varphi ) \sum _{p=0}^\infty c^{lm}_p\, \omega ^{l+2p} \end{aligned}$$

with coefficients bounded by

5.9 $$\begin{aligned} |c^{lm}_p|&\le \sqrt{\frac{4 \pi }{2l+1}}\, \frac{l!}{(2l-1)!!}\, \frac{1}{(l+2p)!}\, \sqrt{\mu (B_1)}\, \Vert Y_{lm}\, \phi ^{lm}\Vert _{L^2(B_1)} \end{aligned}$$

5.10 $$\begin{aligned} |c^{lm}_p|&\le \sqrt{\frac{4 \pi }{2l+1}}\, \frac{l!}{(2l-1)!!}\, \frac{1}{(l+2p+1)!}\, \sqrt{\mu (B_1)}\, \big \Vert \nabla \big ( Y_{lm}\, \phi ^{lm} \big ) \big \Vert _{L^2(B_1)} {.} \end{aligned}$$

### Proof

Since the Fourier transformation preserves angular momentum, it suffices to prove the lemma for fixed *l* and *m*. Moreover, by rotational symmetry it suffices to consider the case $m=0$ (more precisely, the transformation of the *m*-modes under rotations is described by the Wigner *D*-matrix). Hence, expressing the spherical harmonics in terms of Legendre polynomials (see [22,  Eq. 14.30.1]), we obtain

$$\begin{aligned} \phi (x)&= Y_{l0}(\vartheta , \varphi )\, \phi ^{l0}(r) \\ \hat{\phi }(k)&= Y_{l0}(\vartheta ) \sum _{p=0}^\infty c^{l0}_p\, \omega ^{l+2p} = \sqrt{\frac{2l+1}{4 \pi }}\; P_l(k_z) \sum _{p=0}^\infty c^{l0}_p\, |\textbf {k}|^{2p} \end{aligned}$$

where a factor $\omega ^l$ was absorbed into the Legendre polynomial. In order to determine the coefficient $c^{l0}_p$, we differentiate the last equation $l+2p$ times with respect to $k_z$ and evaluate at $k=0$,

$$\begin{aligned} \big (\partial _{k_z}^{l+2p} \hat{\phi }\big )(0) = \begin{pmatrix} l+2p \\ l \end{pmatrix}\, \sqrt{\frac{2l+1}{4 \pi }}\; P_l^{(l)}(0)\; c^{l0}_p\, (2p)! {.} \end{aligned}$$

In order to compute the $l^\text {th}$ derivative of the Legendre polynomial, we must determine the coefficient of its highest power. This can be accomplished with the help of the Rodrigues formula (see [22,  Eq. 18.5.5])5.11and differentiating *l* times gives

$$\begin{aligned} P^{(l)}_l(0) = \frac{(2l)!}{2^l\, l!} = (2l-1)!!{.} \end{aligned}$$

We thus obtain

5.12 $$\begin{aligned} \big (\partial _{k_z}^{l+2p} \hat{\phi }\big )(0) = \sqrt{\frac{2l+1}{4 \pi }} \, (l+2p)!\, \frac{(2l-1)!!}{l!} \, c^{l0}_p {.} \end{aligned}$$

The partial derivative on the left can be estimated by

$$\begin{aligned} \big | \big (\partial _{k_z}^{l+2p} \hat{\phi }\big )(0) \big |&= \bigg | \int _{B_1} (-i z)^{l+2p} \phi (\textbf {x}) \, e^{-i \textbf {k} \textbf {x}}\, \mathrm{d}^3x \bigg | \\&\le \int _{B_1} |\phi (\textbf {x})|\, \mathrm{d}^3x \le \sqrt{\mu (B_1)}\, \Vert \phi \Vert _{L^2(B_1)} {.} \end{aligned}$$

Using this estimate in (5.12) and solving for $c^{l0}_p$ give (5.9).

In order to derive (5.10), we again fix *l* and consider the case $m=0$. Differentiating $\phi$ in the *z*-direction, we obtain

$$\begin{aligned} \widehat{\big ( \partial _z \phi \big )}(k)&= k_z \, \hat{\phi }(k) = k_z\, Y_{l0}(\vartheta ) \sum _{p=0}^\infty c^{l0}_p\, \omega ^{l+2p} = \sqrt{\frac{2l+1}{4 \pi }}\; k_z \,P_l(k_z) \sum _{p=0}^\infty c^{l0}_p\, |\textbf {k}|^{2p} {.} \end{aligned}$$

We now differentiate $l+2p+1$ times with respect to $k_z$ and evaluate at $k=0$,

$$\begin{aligned} \partial _{k_z}^{l+2p+1} \widehat{\big ( \partial _z \phi \big )}(0) = \begin{pmatrix} l+2p+1 \\ l+1 \end{pmatrix}\, \sqrt{\frac{2l+1}{4 \pi }}\; \partial _{k_z}^{l+1} \Big ( k_z \,P_l(k_z) \Big ) \Big |_{k_z=0} \; c^{l0}_p\, (2p)! {.} \end{aligned}$$

Again applying (5.11), we obtain

5.13 $$\begin{aligned} \partial _{k_z}^{l+2p+1} \widehat{\big ( \partial _z \phi \big )}(0)&= \begin{pmatrix} l+2p+1 \\ l+1 \end{pmatrix}\, \sqrt{\frac{2l+1}{4 \pi }}\; \frac{1}{2^l\, l!} \, \frac{(2l)!}{l!} \, (l+1)! \; c^{l0}_p\, (2p)! \nonumber \\&= \sqrt{\frac{2l+1}{4 \pi }}\, (l+2p+1)! \, \frac{(2l-1)!!}{l!} \; c^{l0}_p {.} \end{aligned}$$

On the other hand, the partial derivative on the left can be estimated by

$$\begin{aligned} \big | \big (\partial _{k_z}^{l+2p+1} \widehat{\big ( \partial _z \phi \big )}(0) \big |&= \bigg | \int _{B_1} (-i z)^{l+2p+1} \,\big (\partial _z \phi \big )(\textbf {x}) \, e^{-i \textbf {k} \textbf {x}}\, \mathrm{d}^3x \bigg | \\&\le \int _{B_1} |\nabla \phi (\textbf {x})|\, \mathrm{d}^3x \le \sqrt{\mu (B_1)}\, \Vert \nabla \phi \Vert _{L^2(B_1)} {.} \end{aligned}$$

Combining this estimate with (5.13) gives (5.10). $\square$

Similar to Proposition 4.4, this lemma allows us to estimate each coefficient of the power series in (5.6).

### Proposition 5.4

The coefficients in the power series (5.6) are bounded by

5.14 $$\begin{aligned} |a^{lm}_n| \le d_l\, \frac{\sqrt{E^{lm}(\phi )}}{n!} \qquad \text {with} \qquad d_l := \frac{4 \pi }{\sqrt{6\,(2l+1)}}\, \frac{l!}{(2l-1)!!}{.} \end{aligned}$$

### Proof

This follows immediately by applying Lemma 5.3 to series (5.4) and using (5.7). More precisely, treating the cases of even and odd *n* separately, we obtain

$$\begin{aligned} \big | a^{lm}_{l+2p} \big |&= \frac{1}{2}\, \big |c^{lm}_{1,p}\big | \overset{(5.9)}{\le } \frac{1}{2}\, \frac{\sqrt{6}}{\sqrt{4 \pi }} \, \frac{d_l}{(l+2p)!}\, \sqrt{\mu (B_1)}\, \Vert Y_{lm}\, \phi _1^{lm}\Vert _{L^2(B_1)} \\&= \frac{d_l}{(l+2p)!}\, \frac{1}{\sqrt{2}}\, \Vert Y_{lm}\, \phi _1^{lm}\Vert _{L^2(B_1)} \le \frac{d_l}{(l+2p)!}\, \sqrt{E^{lm}(\phi )} \\ \big | a^{lm}_{l+2p+1} \big |&= \frac{1}{2}\, \big | c^{lm}_{0,p} \big | \overset{(5.10)}{\le } \frac{1}{2}\, \frac{\sqrt{6}}{\sqrt{4 \pi }} \, \frac{d_l}{(l+2p+1)!}\, \sqrt{\mu (B_1)}\, \big \Vert \nabla \big ( Y_{lm}\, \phi _0^{lm} \big ) \big \Vert _{L^2(B_1)} \\&\le \frac{d_l}{(l+2p+1)!}\, \frac{1}{\sqrt{2}}\, \big \Vert \nabla \big ( Y_{lm}\, \phi _0^{lm} \big ) \big \Vert _{L^2(B_1)} \le \frac{d_l}{(l+2p)!}\, \sqrt{E^{lm}(\phi )} {.} \end{aligned}$$

This concludes the proof. $\square$

We now use the same strategy as in Sects. 4.4 and 4.5. We decompose the series $\hat{h}^{lm}_\pm$ in (5.6) into a polynomial of degree *N* and the remainder term,

5.15 $$\begin{aligned} \hat{h}^{lm}_\pm = \hat{h}^{lm}_N + R^{lm}_N \end{aligned}$$

with

$$\begin{aligned} \hat{h}^{lm}_N(\omega ) := \sum _{n=l}^N a^{lm}_n \, \omega ^n \qquad \text {and} \qquad R^{lm}_N(\omega ) := \sum _{n=N+1}^\infty a^{lm}_n \, \omega ^n {.} \end{aligned}$$

Similar to Lemma 4.6, we first show that the remainder term has small $L^2$-norm on the interval $[0, \omega _1]$. The main difference compared to Lemma 4.6 is the additional factor $\omega ^2$ in the integration measure.

### Lemma 5.5

Given $\varepsilon \in [0, 1]$ and $N \in \mathbb {N}_0$, we choose

5.16 $$\begin{aligned} \omega _1 = \bigg ( \frac{\varepsilon ^2}{d_l^2}\, (N+1)!^2\, (2N+5) \bigg )^{\frac{1}{2N+5}} {.} \end{aligned}$$

Then, the remainder term in (5.15) is bounded on $[0, \omega _1]$ by

$$\begin{aligned} \Vert R^{lm}_{\pm \,N}(\omega )\Vert _{L^2([0, \omega _1], \,\omega ^2 \mathrm{d}\omega )} \le 4\varepsilon \,\sqrt{E^{lm}(\phi )} {.} \end{aligned}$$

### Proof

Applying Proposition 5.4, we can estimate the remainder similar to (4.19) by

5.17 $$\begin{aligned} |R^{lm}_N(\omega )|&\le d_l \sum _{n=N+1}^\infty \frac{\omega ^n}{n!} \, \sqrt{E^{lm}(\phi ^\bullet )} \nonumber \\&\le d_l\, c(\omega )\, \frac{\omega ^{N+1}}{(N+1)!} \,\sqrt{E^{lm}(\phi )}\qquad \text {with} \qquad c(\omega ) := \sum _{n=0}^\infty \Big ( \frac{\omega }{N+2} \Big )^n {.} \end{aligned}$$

Choosing $\omega _1$ according to (4.18), we know that for $\varepsilon <1$ for all $\omega \in [0,\omega _1]$,

$$\begin{aligned} \frac{\omega }{N+2} \le \frac{\omega _1}{N+2} \le \frac{\big ( (N+1)!^2\, (2N+5) \big )^{\frac{1}{2N+5}}}{N+2} \le \frac{3}{4}{,} \end{aligned}$$

where the last inequality is verified by direct inspection and using the Stirling formula. Therefore, the geometric series in (5.17) converges and is bounded by four,

$$\begin{aligned} |R^{lm}_N(\omega )| \le 4 d_l\, \frac{\omega ^{N+1}}{(N+1)!} \,\sqrt{E^{lm}(\phi )} {.} \end{aligned}$$

Using this pointwise bound, the $L^2$-norm can be estimated by

$$\begin{aligned} \Vert R^{lm}_{\pm \,N}(\omega )\Vert ^2_{L^2([0, \omega _1],\, \omega ^2 \mathrm{d}\omega )}&\le 16 \,d_l^2\, E^{lm}(\phi ) \int _0^{\omega _1} \frac{\omega ^{2N+4}}{(N+1)!^2}\, \mathrm{d}\omega \\&\le \frac{16 \,d_l^2\, E^{lm}(\phi )}{(N+1)!^2\, (2N+5)} \,\omega _1^{2N+5} {,} \end{aligned}$$

giving the result. $\square$

Now, we can estimate each Taylor coefficient by using the method in Lemma 4.5. The following result is the analog of Proposition 4.7.

### Proposition 5.6

Assume that for any given $l \in \mathbb {N}_0$, $m \in \{-l, \ldots , l\}$ and $\varepsilon \in (0,1]$,

$$\begin{aligned} E^{lm}(\phi _-) \le \varepsilon ^2\, E^{lm}(\phi ) {.} \end{aligned}$$

Then, the series coefficients in (5.6) are bounded by

$$\begin{aligned} |a^{lm}_n| \le 25\, \max \Big (d_l, d_l^{\frac{2l+3}{2l+5}} \Big ) \,\frac{1}{\sqrt{2n+1}} \,\frac{4^n}{n!}\, \varepsilon ^{\frac{2}{2n+5}} \, \sqrt{E^{lm}(\phi )}{.} \end{aligned}$$

### Proof

Given $N \in \mathbb {N}_0$, we choose $\omega _1$ as in (5.16). Decomposing the function $\hat{h}^{lm}_-$ according to (5.15), the $L^2$-norm of the remainder is bounded according to Lemma 5.5. Combining this fact with Lemma 5.2, we obtain

$$\begin{aligned}&\Vert \hat{h}^{lm}_{N}(\omega )\Vert _{L^2([0, \omega _1],\, \omega ^2 \mathrm{d}\omega )} = \big \Vert \hat{h}_-^{lm} - R_{N}^{lm} \big \Vert _{L^2([0, \omega _1],\, \omega ^2 \mathrm{d}\omega )} \\&\quad \le \big \Vert \hat{h}_-^{lm} \big \Vert _{L^2([0, \omega _1],\, \omega ^2 \mathrm{d}\omega )} + \big \Vert R_{-\,N}^{lm} \big \Vert _{L^2([0, \omega _1],\, \omega ^2 \mathrm{d}\omega )}\\&\quad \le \sqrt{2\pi ^2\, E^{lm}(\phi _-)} + \Vert R_{-\,N}^{lm}\Vert _{L^2([0, \omega _1])} \\&\quad \le \varepsilon \, \sqrt{2\pi ^2\, E^{lm}(\phi )} + 4\varepsilon \,\sqrt{E^{lm}(\phi )} \le 9 \varepsilon \, \sqrt{E^{lm}(\phi )} {.} \end{aligned}$$

Applying Lemma 4.5 to the polynomial ${\mathcal {P}}(\omega ):= \omega \,\hat{h}^{lm}_N(\omega )$ gives the bound

$$\begin{aligned} |a^{lm}_N|&\le \frac{1}{\sqrt{\omega _1}}\, \bigg ( \frac{4}{\omega _1} \bigg )^{N+1}\, \Vert {\mathcal {P}}\Vert _{L^2([0,\omega _1], \mathrm{d}\omega )} \\&= \frac{1}{\sqrt{\omega _1}}\, \bigg ( \frac{4}{\omega _1} \bigg )^{N+1}\, \Vert \hat{h}^{lm}_{N}(\omega )\Vert _{L^2([0, \omega _1],\, \omega ^2 \mathrm{d}\omega )} \\&\le 4^{N+1}\, \omega _1^{-N-\frac{3}{2}} \,6 \varepsilon \, \sqrt{E^{lm}(\phi )} \\&\le 9\cdot 4^{N+1}\, d_l^{\frac{2N+3}{2N+5}} \,\varepsilon ^{\frac{2}{2N+5}} \, (N+1)!^{-\frac{2N+3}{2N+5}}\, (2N+5)^{-\frac{2N+3}{4N+10}}\, \sqrt{E^{lm}(\phi )} {.} \end{aligned}$$

The result follows asymptotically from the Stirling formula and for small values of *n* directly by numerical evaluation. $\square$

Now, we are ready to extend Proposition 4.8 to the $3+1$-dimensional setting.

### Proposition 5.7

Assume that for any given $l \in \mathbb {N}_0$, $m \in \{-l, \ldots , l\}$ and $\varepsilon \in (0,1]$, the energy of the negative-frequency component is bounded in terms of the total energy by

$$\begin{aligned} E^{lm}(\phi _-) \le \varepsilon ^2\, E^{lm}(\phi ) {.} \end{aligned}$$

Then, the initial data in momentum space is bounded pointwise for all $\omega \in \mathbb {R}^+$ by

$$\begin{aligned} \big |\hat{h}^{lm}(\omega )\big | \le 25\, \max \Big (d_l, d_l^{\frac{2l+3}{2l+5}} \Big )\, \sqrt{E^{lm}(\phi )} \; \big ( 4\omega \big )^{-\frac{3}{2}}\, g_l\big (\omega , \varepsilon \big ) {,} \end{aligned}$$

where $g_l$ is the series

$$\begin{aligned} g_l(\omega , \varepsilon ) := \sum _{n=l}^\infty \frac{1}{\sqrt{2n+1}}\, \frac{(4 \omega )^{n+\frac{3}{2}}}{n!}\, \varepsilon ^{\frac{2}{2n+5}} {.} \end{aligned}$$

The series $g_l$ in (4.20) differ from the corresponding series *g* in (4.20) in two points: The sum begins at $n=l$ (which makes the series smaller) and the power of $\varepsilon$ is $2/(2n+5)$ instead of $2/(2n+3)$ (which makes the series larger). The different power comes about as a consequence of the factor $\omega ^2$ in the integration measure in (5.8).

The remaining task is to estimate the series $g_l$. All the methods developed in the $1+1$-dimensional setting can be adapted to the new series in (4.20). A simple method for getting the connection is to estimate $g_l$ by

5.18 $$\begin{aligned} g_l(\omega , \varepsilon )&= \sum _{n=l}^\infty \frac{1}{\sqrt{2n+1}}\, \frac{(4 \omega )^{n+\frac{3}{2}}}{n!}\, \big ( \varepsilon ^{\frac{2n+3}{2n+5}} \big )^{\frac{2}{2n+3}} \nonumber \\&\le \sum _{n=0}^\infty \frac{1}{\sqrt{2n+1}}\, \frac{(4 \omega )^{n+\frac{3}{2}}}{n!}\, \big ( \varepsilon ^{\frac{2l+3}{2l+5}} \big )^{\frac{2}{2n+3}} = g\big ( \omega , \varepsilon ^{\frac{2l+3}{2l+5}} \big ) {.} \end{aligned}$$

This method is not quite optimal but seems sufficient for most applications. For more refined estimates, one needs to reconsider the constructions in Sects. 4.9–4.14 with modified exponents. For brevity, we do not enter the details here.

We conclude this section with two theorems. We begin with an estimate for each angular momentum mode, obtained by combining Proposition 5.7 with estimate (5.18) and Proposition 4.24.

### Theorem 5.8

Let $\phi (t,x)$ be a solution of the $3+1$-dimensional scalar wave equation which at some time $t_0$ is supported inside a ball of radius $r>0$,

$$\begin{aligned} {{\,\mathrm{supp}\,}}\phi (t_0, .) \in B_r(0) {.} \end{aligned}$$

Assume that for any given $l \in \mathbb {N}_0$, $m \in \{-l, \ldots , l\}$ and $\varepsilon \in (0,1]$, the energy of the negative-frequency component is bounded in terms of the total energy by

$$\begin{aligned} E^{lm}(\phi ) \le \varepsilon ^2\, E^{lm}(\phi ) {.} \end{aligned}$$

Then, there is an a priori estimate for the momentum distribution of $\phi$ of the form

$$\begin{aligned} \big |k\,\hat{\phi }^{lm}(k) \big | + \big | \partial _t \hat{\phi }^{lm}(k) \big | \le R_l\big (\varepsilon , r \,|k| \big ) \,\sqrt{r^3\,E^{lm}(\phi )}{,} \end{aligned}$$

where the function $R_l$ is given by

$$\begin{aligned} R_l(\varepsilon , \omega ) = c\, \max \Big (d_l, d_l^{\frac{2l+3}{2l+5}} \Big )\, \exp \bigg ( \frac{3}{2} \beta ^2 + \sqrt{2b} \,\Big ( \frac{1}{2\beta } - \beta \Big ) \bigg ) \, \sqrt{\frac{e^{-\nu }}{\sqrt{\nu }}\, \text {Erfi}(\nu ) } {.} \end{aligned}$$

Here, *c* is a numerical constant (which is independent of *l*), $d_l$ are the constants in (5.14), and $\beta$ and $\nu$ are given implicitly by

$$\begin{aligned} 2 \,\sqrt{\frac{2l+3}{2l+5}\, |\log \varepsilon |}&= 3\, \beta + 8 \omega \,\beta \, e^{\beta ^2} \\ \nu&= 4 \omega \,e^{\beta ^2} {.} \end{aligned}$$

Finally, by combining the estimates for each angular mode and summing over the modes, we derive an estimate for a general solution to the $3+1$-dimensional wave equation.

### Theorem 5.9

Assume that for $\varepsilon \in (0,1]$, the energy of the negative-frequency component is bounded in terms of the total energy by

$$\begin{aligned} E(\phi _-) \le \varepsilon ^2\, E(\phi ) {.} \end{aligned}$$

Then, the $L^2$-norm of the spatial Fourier transform on a sphere of radius $\omega$ is bounded for all $\omega \in \mathbb {R}^+$ by

$$\begin{aligned} \int _{S^2} \big |\omega \,\hat{\phi }(\vartheta ,\phi ,\omega )\big |^2\, \mathrm{d}\mu _S^2(\vartheta , \varphi ) \le 625 \,d_0^{\frac{10}{3}}\, C\, E(\phi ) \; \big ( 4\omega \big )^{-\frac{6}{2}}\, g^2_0\big (\omega , \varepsilon \big ) {,} \end{aligned}$$

where *C* is the constant

$$\begin{aligned} C := \sum _{l=0}^\infty (2l+1) \,d_l^{\frac{4l+6}{2l+5}}<\infty \end{aligned}$$

(and the $d_l$ are again given by (5.14)).

### Proof

In order to simplify the calculations, we observe that $d_l>1$ only for $l=\{0,1,2,3\}$ and thus

$$\begin{aligned} \max \Big (d_l, d_l^{\frac{2l+3}{2l+5}} \Big ) \le d_0^{\frac{5}{3}} \,d_l^{\frac{2l+3}{2l+5}} \qquad \text {for all } l\in \mathbb {N}_0{.} \end{aligned}$$

Using this estimate in the statement of Proposition 5.7, where we choose parameters $\varepsilon _{lm}$ such that $E^{lm}(\phi _-) = \varepsilon _{lm}^2\, E^{lm}(\phi )$, we obtain

$$\begin{aligned} \int _{S^2}&\big |\omega \,\hat{\phi }(\vartheta ,\varphi ,\omega )\big |^2 \,\mathrm{d}\mu _{S^2} =\sum _{l=0}^\infty \sum _{m=-l}^l \big |\hat{h}^{lm}(\omega )\big |^2\\&\le 625\, d_0^{\frac{10}{3}}\, \sum _{l=0}^\infty \sum _{m=-l}^l d_l^{\frac{4l+6}{2l+5}} \, E^{lm}(\phi )\big ( 4\omega \big )^{-\frac{6}{2}}\, g^2_l\big (\omega , \varepsilon _{lm} \big ) {.} \end{aligned}$$

Along the lines of the proof of Theorem 4.9, we use that the relations

$$\begin{aligned} E^{lm}(\phi )= \delta _{lm}\, E(\phi ) \qquad \text {and} \qquad E^{lm}(\phi _-)= \varepsilon ^2_{lm}\, E^{lm}(\phi ) \end{aligned}$$

imply that for all *l*, *m* with $\varepsilon _{lm}>\varepsilon$, the inequality $\delta _{lm}\le \frac{\varepsilon ^2}{\varepsilon _{lm}^2}$ holds. We thus obtain

$$\begin{aligned}&\int _{S^2} \big |\omega \,\hat{\phi }(\vartheta ,\varphi ,\omega )\big |^2 \,\mathrm{d}\mu _{S^2} \le 625\, d_0^{\frac{10}{3}}\, E(\phi )\, \sum _{l=0}^\infty \sum _{m=-l}^l d_l^{\frac{4l+6}{2l+5}} \,\delta _{lm}\big ( 4\omega \big )^{-\frac{6}{2}}\, g^2_l\big (\omega , \varepsilon _{lm} \big )\\&\quad \le 625\, d_0^{\frac{10}{3}}\, E(\phi )\, \left( \sum _{\varepsilon _{lm}\le \varepsilon }d_l^{\frac{4l+6}{2l+5}} \,\big ( 4\omega \big )^{-\frac{6}{2}}\, g^2_l\big (\omega , \varepsilon \big ) + \sum _{\varepsilon _{lm}>\varepsilon }d_l^{\frac{4l+6}{2l+5}} \,\big ( 4\omega \big )^{-\frac{6}{2}}\, g^2_l\big (\omega , \varepsilon _{lm} \big )\frac{\varepsilon ^2}{\varepsilon _{lm}^2}\right) . \end{aligned}$$

For all the modes with $\varepsilon _{lm}\le \varepsilon$, we used that in this case, $g_l(\omega ,\varepsilon _{lm})<g_l(\omega ,\varepsilon )$ for all *l*, *m* and that $\delta _{lm}\le 1$ due to Lemma 5.2. With the same argument as in the proof of Theorem 4.9, it follows that $\frac{\partial }{\partial \varepsilon _{lm}} \bigg ( g_l^2(\omega ,\varepsilon _{lm}) \,\frac{\varepsilon ^2}{\varepsilon _{lm}^2} \bigg )<0$ for $\varepsilon \in [0,1)$ and thus

$$\begin{aligned} g_l^2(\omega ,\varepsilon _{lm}) \,\frac{\varepsilon ^2}{\varepsilon _{lm}^2}\le g_l^2(\omega ,\varepsilon ) \qquad \text {for all } l,m{,} \end{aligned}$$

giving rise to the estimate

$$\begin{aligned} \int _{S^2} \big |\omega \,\hat{\phi }(\vartheta ,\varphi ,\omega )\big |^2 \,\mathrm{d}\mu _{S^2}&\le 625\, d_0^{\frac{10}{3}}\, E(\phi )\, \sum _{l=0}^\infty \sum _{m=-l}^l d_l^{\frac{4l+6}{2l+5}} \,\big ( 4\omega \big )^{-\frac{6}{2}}\, g^2_l\big (\omega , \varepsilon \big )\\&\le 625\, d_0^{\frac{10}{3}}\, E(\phi )\big ( 4\omega \big )^{-\frac{6}{2}}\, g^2_0\big (\omega , \varepsilon \big )\, \sum _{l=0}^\infty \sum _{m=-l}^ld_l^{\frac{4l+6}{2l+5}} {,} \end{aligned}$$

where in the last step we used that $g_l(\omega ,\varepsilon )\le g_0(\omega ,\varepsilon )$ for all $l\in \mathbb {N}$. Carrying out the sum over *m*, we obtain the series

$$\begin{aligned} \sum _{l=0}^\infty (2l+1) \, d_l^{\frac{4l+6}{2l+5}} {.} \end{aligned}$$

Using (5.14) and applying Stirling’s formula to each term of the resulting series

$$\begin{aligned} \sum _{l=0}^\infty (2l+1)^{\frac{2}{2l+5}} \,\bigg (\frac{8 \pi ^2}{3}\, \Big ( \frac{l!}{(2l-1)!!}\Big )^2 \bigg )^{\frac{2l+3}{2l+5}} {,} \end{aligned}$$

one sees that this series converges absolutely. This completes the proof. $\square$

## Appendix A. Alternative Derivation of the Integral Representation

In this appendix, we give an alternative derivation of the integral representation of the solutions of the Goursat problem (4.36). The method is by direct computation using the series representation of the Bessel function $J_0$.

### Lemma 5.10

Let *g*(*a*) be a power series of the form

$$\begin{aligned} g(a) = \sum _{n=0}^\infty c_n\, e^{(2n+3)a} {.} \end{aligned}$$

Then, for all $a,b>0$,

A.1 $$\begin{aligned} \sum _{n=0}^\infty c_n\, e^{(2n+3)a - \frac{b}{2n+3}} = \int _{-\infty }^a J_0 \Big ( 2 \,\sqrt{(a-\tau )\, b}\, \Big )\, g'(\tau )\, \mathrm{d}\tau {.} \end{aligned}$$

**Proof **The Bessel function $J_0$ has the power expansion (see [22,  Eq. 10.2.2])

$$\begin{aligned} J_0(z) = \sum _{\ell =0}^\infty \frac{(-1)^\ell }{(\ell !)^2}\, \Big ( \frac{z^2}{4} \Big )^\ell {.} \end{aligned}$$

Denoting the right side of (A.1) by *T*(*a*, *b*), we obtain

$$\begin{aligned} T(a,b) \,&:= \int _{-\infty }^a J_0 \Big ( 2 \,\sqrt{(a-\tau )\, b} \,\Big )\, g'(\tau )\, \mathrm{d}\tau \\&= \int _{-\infty }^a \sum _{\ell ,n=0}^\infty \frac{(-1)^\ell }{(\ell !)^2}\, \big ( (a-\tau )\, b \big )^\ell \; (2n+3)\, c_n\, e^{(2n+3)\,\tau } \, \mathrm{d}\tau {.} \end{aligned}$$

Introducing the new integration variable $\xi =(2n+3)(a-\tau )$ gives

$$\begin{aligned} T(a,b)&= \int _0^\infty \sum _{\ell ,n=0}^\infty \frac{(-1)^\ell }{(\ell !)^2}\, \Big ( \frac{b}{2n+3}\, \xi \Big )^\ell \; (2n+3)\, c_n\, e^{-\xi + (2n+3)\,a} \, \frac{\mathrm{d}\xi }{2n+3} \\&= \sum _{\ell ,n=0}^\infty \frac{(-1)^\ell }{(\ell !)^2}\, \Big ( \frac{b}{2n+3} \Big )^\ell \; c_n\, e^{(2n+3)\,a} \, \int _0^\infty \xi ^\ell \, e^{-\xi } \,\mathrm{d}\xi \\&= \sum _{\ell ,n=0}^\infty \frac{(-1)^\ell }{\ell !}\, \Big ( \frac{b}{2n+3} \Big )^\ell \; c_n\, e^{(2n+3)\,a}\\&= \sum _{n=0}^\infty \exp \Big (-\frac{b}{2n+3} \Big )\; c_n\, e^{(2n+3)\,a} {,} \end{aligned}$$

where in the last step we carried out the $\ell$-series to obtain an exponential. $\square$

## Footnotes

1 In order to avoid confusion for readers with a more mathematical background, we note that, here and throughout the paper, by *frequency* we always refer to oscillations in the time variable. (In contrast, wave vectors in the spatial variables are referred to as *momenta*.)

2 We define the Fourier transform with a factor of one and the inverse Fourier transform with a factor of $1/(2\pi )$.
